# Newly identified antimicrobial activity of an 8-hydroxyquinoline-based ionophore against multidrug-resistant *Enterococcus faecium* and *Staphylococcus aureus*

**DOI:** 10.1093/jac/dkag186

**Published:** 2026-06-03

**Authors:** Gen Li, Ibrahim M El-Deeb, Hayden G Whyte, Mark A T Blaskovich, Mark J Walker, Mark von Itzstein, David M P De Oliveira

**Affiliations:** Australian Infectious Diseases Research Centre, Institute for Molecular Bioscience, The University of Queensland, Brisbane, QLD 4072, Australia; Centre for Superbug Solutions, Institute for Molecular Bioscience, The University of Queensland, Brisbane, QLD 4072, Australia; Institute for Biomedicine and Glycomics, Griffith University, Southport, QLD 4222, Australia; Australian Infectious Diseases Research Centre, Institute for Molecular Bioscience, The University of Queensland, Brisbane, QLD 4072, Australia; Centre for Superbug Solutions, Institute for Molecular Bioscience, The University of Queensland, Brisbane, QLD 4072, Australia; Centre for Superbug Solutions, Institute for Molecular Bioscience, The University of Queensland, Brisbane, QLD 4072, Australia; Community for Open Antimicrobial Drug Discovery, Institute for Molecular Bioscience, The University of Queensland, Brisbane, QLD 4072, Australia; Australian Infectious Diseases Research Centre, Institute for Molecular Bioscience, The University of Queensland, Brisbane, QLD 4072, Australia; Centre for Superbug Solutions, Institute for Molecular Bioscience, The University of Queensland, Brisbane, QLD 4072, Australia; Institute for Biomedicine and Glycomics, Griffith University, Southport, QLD 4222, Australia; Australian Infectious Diseases Research Centre, Institute for Molecular Bioscience, The University of Queensland, Brisbane, QLD 4072, Australia; Centre for Superbug Solutions, Institute for Molecular Bioscience, The University of Queensland, Brisbane, QLD 4072, Australia

## Abstract

**Background and objectives:**

*Enterococcus faecium* and *Staphylococcus aureus* are opportunistic bacterial pathogens with a demonstrated capacity to develop antimicrobial resistance and cause serious life-threatening infections, underscoring the urgent need for new therapeutic options.

**Methods:**

Here, we have synthesized and characterized the activities of an 8-hydroxyquinoline–based ionophore antibiotic (ionophoroantibiotic; IP antibiotic), designated ‘IP-antibiotic 12.’

**Results:**

Using multidrug-resistant strains of *E. faecium* and *S. aureus*, *in vitro* investigations revealed that IP-antibiotic 12 exhibits bactericidal activity, demonstrates a low propensity for resistance emergence, increases the susceptibility of particular strains to select antibiotics, possesses a favorable toxicity profile, and dysregulates bacterial metal homeostasis. IP-antibiotic 12 demonstrated therapeutic efficacy against multidrug-resistant *S. aureus* skin infection, as a direct-acting topical antimicrobial and antibiotic adjunct when co-administered with oral linezolid. Interestingly, it was not efficacious in murine models of systemic and pulmonary infection.

**Conclusions:**

These results highlight the potential of IP-antibiotic 12 as a novel therapeutic against multidrug-resistant gram-positive bacteria and provide a foundation for the development of next-generation IP-antibiotics with enhanced *in vivo* therapeutic efficacy.

## Introduction

*Enterococcus faecium* and *Staphylococcus aureus* are commensal pathogens of significant clinical concern, causing infections that range from minor superficial wound infections to life-threatening systemic diseases. Over recent decades, both species have acquired extensive antimicrobial resistance (AMR), including against antibiotics of last resort.^1–4^ Notably, *E. faecium* and *S. aureus* are among the leading gram-positive pathogens responsible for AMR-attributed and -associated deaths.^5,6^ The urgent need for new treatments targeting multidrug-resistant (MDR) strains of *E. faecium* and *S. aureus* is underscored by their classification as high-priority pathogens for research and development by the World Health Organization.^7^

Despite the urgent need for new antibiotics, discovery efforts have steadily declined due to the high-cost, lengthy timelines, and substantial risk associated with *de novo* drug development.^8–11^ As a result, drug repurposing has emerged as a promising strategy to accelerate the identification of new antimicrobial therapies.^12,13^ Among the candidates for repurposing are ionophores, a diverse class of natural and synthetic ion transporters, which have demonstrated broad-spectrum antimicrobial activity in humans.^14^ The combination of zinc and the 8-hydroxyquinoline (8-HQ)–based ionophore 5,7-dichloro-2-[*N*,*N-*(dimethylamino)methyl]quinolin-8-ol (PBT2), originally developed for the treatment of neurodegenerative diseases, has been shown to exhibit effective antibacterial activity against MDR *E. faecium* and *S. aureus*, both as a direct antimicrobial and antibiotic potentiator *in vitro* and against wound infection *in vivo*, respectively. PBT2 exerts antimicrobial activity through dysregulation of bacterial transition metal ion homeostasis, leading to disruption of virulence determinants and metabolic networks that together diminish pathogenicity and resistance capacity.^4,14–16^ In Phase II human trials, PBT2 exhibited a favourable safety and tolerability profile, with once-daily oral dosing at 250 mg/day shown to be safe for up to 24 months in Alzheimer’s patients^17^ and up to 26 weeks in Huntington’s patients,^18^ attesting to the safety of 8-HQ–based ionophores.

Here, we have explored the capacity of twelve different IP-antibiotic candidates to inhibit the growth of MDR *E. faecium* and *S. aureus*. Our screening results showed that IP-antibiotic 12 exerted direct antimicrobial and antibiotic-potentiating activity against MDR *E. faecium* and *S. aureus in vitro* in the absence of exogenous zinc. Although IP-antibiotic 12 was originally synthesized by Carissimi et al,^19^ our study herein is the first to investigate and report on the novel antibacterial properties of this compound. We show that IP-antibiotic 12 exhibited a low propensity for resistance development, enhanced the activity of select pathogen–drug combinations, and disrupted bacterial metal homeostasis *in vitro*. *In vivo*, IP-antibiotic 12 was efficacious both as a direct-acting antimicrobial and as an antibiotic potentiator against vancomycin-resistant *S. aureus* (VRSA) skin infection in mice. These findings support the continued development of 8-HQ–based ionophores as a novel class of direct-acting IP-antibiotic agents to address the growing threat of AMR.

## Materials and methods

### General synthetic methods

**Figure 1.**
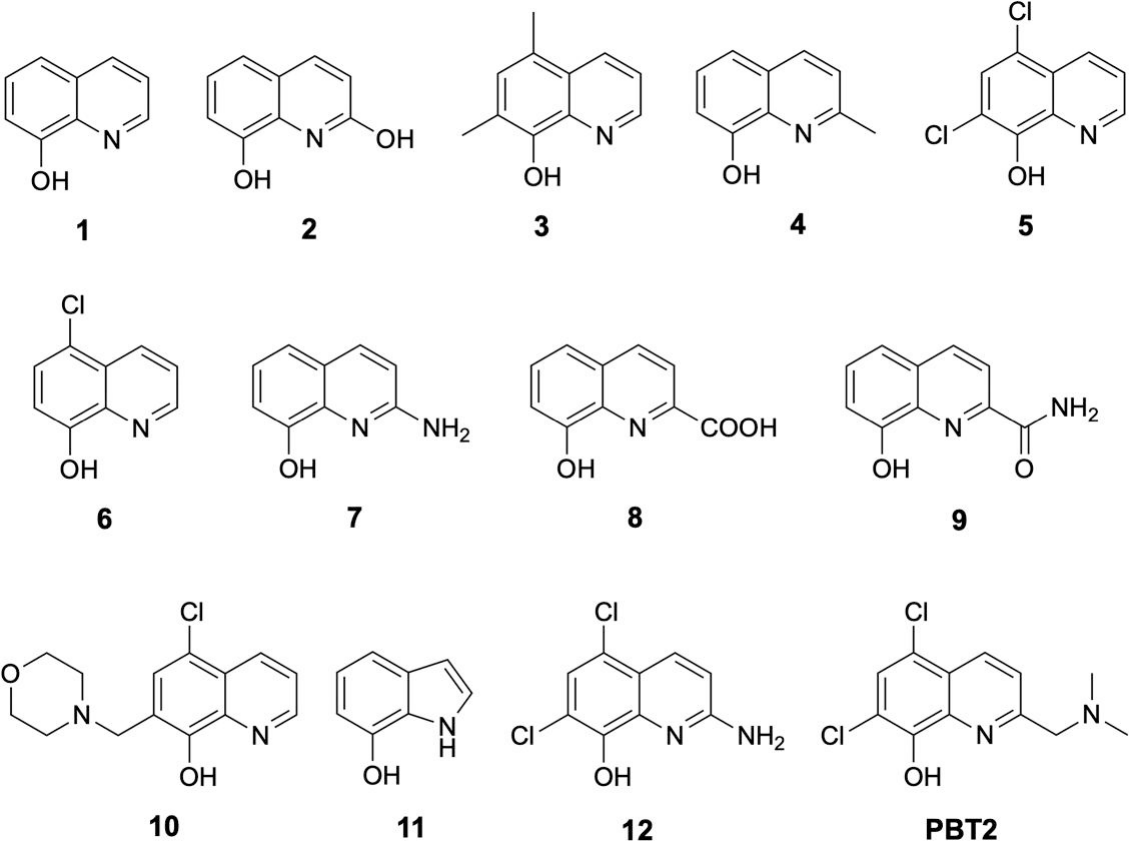
Structures of tested IP-antibiotic candidates 1–12 and reference ionophore PBT2. Candidates 1–11 were purchased from Sigma-Aldrich (IUPAC names detailed in Table S2), while candidate 12 was chemically synthesized (refer to ‘General synthetic methods’ under Materials and Methods section). PBT2 was supplied from Alterity Therapeutics. All compounds were dissolved in 100% DMSO prior to MIC testing.

Reagents and dry solvents purchased from commercial sources were used without further purification. Anhydrous reactions were carried out under an atmosphere of argon, using oven-dried glassware. IP-antibiotics 1–11 were purchased from Sigma-Aldrich (Table S2), while IP-antibiotic 12 was chemically synthesized, and the detailed experimental synthetic methods used are explained below. Reactions were monitored using thin-layer chromatography (TLC) on aluminium plates pre-coated with Silica Gel 60 F254 (E. Merck). Developed plates were observed under UV light at 254 nm. Flash chromatography was performed on Silica Gel 60 (0.040–0.063 mm). ^1^H and ^13^C NMR spectra were recorded at 400 and 100 MHz, respectively, on a BrukerAvance 400 MHz spectrometer. Chemical shifts (δ) were reported in parts per million, relative to the residual solvent peak as internal reference [CDCl_3_: 7.26 (s) for ^1^H, 77.0 (t) for ^13^C; *d*_6_-DMSO: 2.50 (pent) and 3.33 (s) for ^1^H, 39.51 (hept) for ^13^C]. Low-resolution mass spectra (LRMS) were recorded in electrospray ionization mode on a Bruker Daltonics Esquire 3000 ESI spectrometer using positive and negative ionization modes. High-resolution liquid chromatography mass spectrometry (LCMS) analysis was performed using a Vanquish Flex ultra-high-performance liquid chromatography system (UHPLC) coupled with a Thermo Fisher Orbitrap Exploris 120 mass spectrometer. Separation was achieved on a Kinetex XB-C18 100 Å column (100 × 2.1 mm, 2.6 µm). Samples were dissolved in acetonitrile and filtered prior to injection using 0.45 µm polytetrafluoroethylene (PTFE) filters. Ionization was achieved by H-ESI, and analysis was performed in both positive and negative modes. The detailed synthetic steps below employ more advanced, higher yielding, and milder reaction conditions compared to those previously reported.^19^ The purity of IP-antibiotic 12 was judged to be >95% by ^1^H NMR and ^13^C NMR spectroscopy (Figure S6). Structures of all referenced compounds are shown in Figure 1.

IP-antibiotic 12 is a simplified analogue of PBT2 with a free amine group at position 2 of the quinoline ring, in place of the *N,N*-dimethylaminomethyl side chain in PBT2. The design concept for this new template is to directly attach the side chain amino group in PBT2 to C2 of the 8-HQ nucleus and assess the effect of this change on metal binding capacity and hence the antibacterial activity of the compound relative to the parent ionophore PBT2. In addition to IP-antibiotic 12, 11 other commercially available 8-HQ derivatives (except for IP-antibiotic 11, which is an indole derivative) were tested for comparison against PBT2 and IP-antibiotic 12. The effect of the amine substituent at position 2 in IP-antibiotic 12 was explored by comparing the antibacterial activity of this compound to that of PBT2 and the commercially available ionophores. Finally, to test for the importance of the two chloro-substituents at positions 5 and 7 of the 8-HQ ring, IP-antibiotic 7 (that lacks these two chloride atoms while having a 2-amine substituent, similar to IP-antibiotic 12) was tested and compared to IP-antibiotic 12. The results showed that both the dichloro-substituents and the 2-amine substituent are essential for potent antibacterial activity.

#### 8-(Benzyloxy)-5,7-dichloro-2-methylquinoline (compound 13)

Potassium carbonate (8.47 g; 61.3 mmol) and tetrabutylammonium iodide (80.9 mg; 0.22 mmol) were added to a stirred solution of 5,7-dichloro-2-methylquinolin-8-ol (10.0 g; 43.8 mmol) in *N,N*-dimethylformamide (DMF) (100 mL). Benzyl bromide (6.78 mL; 57.0 mmol) was then added, and the mixture was left to stir at room temperature for 42 h under a nitrogen atmosphere. Upon confirmation of reaction completion by TLC, the crude mixture was concentrated and purified by silica gel chromatography using gradient solvent system of hexane/ethyl acetate (4% → 10%) to yield pure compound 13 (12.0 g; 86% yield). ^1^H NMR (400 MHz, CDCl_3_): δ 2.81 (s, 3H, CH_3_), 5.46 (s, 2H, Bn-CH_2_), 7.30–7.42 (m, 4H), 7.55 (s, 1H), 7.60–7.65 (m, 2H), 8.38 (d, *J* = 8.7 Hz, 1H); ^13^C NMR (101 MHz, CDCl_3_): δ 25.56 (CH_3_), 76.58 (Bn-CH_2_), 122.85, 124.55, 126.08, 126.82, 126.90, 128.16, 128.29, 128.78, 133.34, 137.20, 143.37, 149.78, 159.90; LRMS [C_17_H_13_Cl_2_NO] (*m/z*): (+ve ion mode) 339.8 [M + Na]^+^; HRMS (API) (*m/z*): [M + H]^+^ calculated for C_17_H_13_Cl_2_NO, 318.0447; found, 318.0444 (Figure S7).

#### 8-(Benzyloxy)-5,7-dichloroquinoline-2-carboxylic acid (compound 14)

Selenium dioxide (9.85 g; 88.8 mmol) was added to a stirred solution of compound 13 (9.60 g; 23.18 mmol) in pyridine (85 mL) and stirred at 115°C under a nitrogen atmosphere for 5 h, then cooled to room temperature, and filtered through a pad of Celite. The filtrate was concentrated under vacuum, then reconcentrated from toluene, and the residue was dissolved in dichloromethane under sonication and washed with 1.0 M sodium hydroxide. The resulting precipitate was removed by filtration, washed with water, then suspended in water/methanol (1:1), acidified with 2.0 M hydrochloric acid, and refiltered. The aqueous phase of the initial filtrate was also acidified and refiltered, and the precipitates were combined and dried under vacuum to yield pure compound 14 at (9.87 g; 94% yield). ^1^H NMR (400 MHz, DMSO-*d*_6_): δ 5.59 (s, 2H, Bn-CH_2_), 7.27–7.44 (m, 3H), 7.55–7.68 (m, 2H), 8.04 (s, 1H), 8.27 (d, *J* = 8.7 Hz, 1H), 8.69 (d, *J* = 8.8 Hz, 1H); ^13^C NMR (101 MHz, DMSO-*d*_6_): δ 76.76 (Bn-CH_2_), 122.83, 125.42, 126.73, 126.98, 128.70, 128.72, 129.26, 129.59, 135.26, 137.27, 142.62, 150.01, 150.75, 166.36 (COOH); LRMS [C_17_H_11_Cl_2_NO_3_] (*m/z*): (+ve ion mode) 370.0 [M + Na]^+^; HRMS (API) (*m/z*): [M + H]^+^ calculated for C_17_H_11_Cl_2_NO_3,_ 348.0189; found, 348.0185 (Figure S8).

#### *Tert*-butyl (8-(benzyloxy)-5,7-dichloroquinolin-2-yl)carbamate (compound 15)

*N,N*-diisopropylethyl-amine (8.80 mL; 50.6 mmol) and diphenylphosphoryl azide (10.9 mL; 50.60 mmol) were added to a stirred solution of compound 14 (9.87 g, 28.37 mmol) in *tert*-butanol (150 mL), and stirred at 90°C under an argon atmosphere for 18 h. The reaction mixture was then cooled to room temperature, concentrated under a vacuum, and purified by silica gel chromatography using a gradient solvent system of hexane/ethyl acetate (0% → 4%) to yield pure compound 15 (11.53 g, 97% yield). ^1^H NMR (400 MHz, CDCl_3_): δ 1.49 (s, 9H, Boc-3CH_3_), 5.31 (s, 2H, Bn-CH_2_), 7.27–7.39 (m, 3H), 7.43–7.57 (m, 3H), 8.22 (s, 1H, NH), 8.30 (d, *J* = 9.3 Hz, 1H), 8.44 (d, *J* = 9.2 Hz, 1H); ^13^C NMR (101 MHz, CDCl_3_): δ 28.15 (Boc-3CH_3_), 76.06 (Bn-CH_2_), 81.62 (OCCH_3_), 113.68, 123.50, 125.58, 126.38, 127.67, 128.09, 128.17, 128.27, 135.83, 137.08, 142.38, 148.70, 152.19 (CO), 152.29; LRMS [C_21_H_20_Cl_2_N_2_O_3_] (*m/z*): (+ve ion mode) 440.7 [M + Na]^+^; HRMS (API) (*m/z*): [M + H]^+^ calculated for C_21_H_20_Cl_2_N_2_O_3_, 419.0924; found, 419.0920 (Figure S9).

#### 8-(Benzyloxy)-5,7-dichloroquinolin-2-amine (compound 16)

To a solution of compound 15 (5.0 g, 12 mmol) in dichloromethane (DCM, 50 mL) at 0°C was slowly added TFA (10 mL). The mixture was stirred in ice bath and then allowed to warm gradually to room temperature. The reaction mixture was stirred overnight at room temperature, diluted with DCM (100 mL), and washed with saturated aqueous NaHCO_3_ (100 mL × 3). The organic layer was then separated, washed with brine (50 mL), dried over anhydrous NaSO_4_ and concentrated under vacuum to yield the amine compound 16 (3.73 g, 98% yield). ^1^H NMR (400 MHz, DMSO-*d*_6_): δ 5.36 (s, 2H), 6.92 (d, *J* = 9.2 Hz, 1H), 6.99 (s, 2H), 7.29–7.42 (m, 4H), 7.52–7.58 (m, 2H), 8.09 (d, *J* = 9.1 Hz, 1H); ^13^C NMR (101 MHz, DMSO*-d*_6_): δ 75.16, 114.30, 120.55, 121.53, 125.73, 126.16, 128.39, 128.65, 128.84, 134.02, 137.85, 144.17, 147.41, 158.86; HRMS (API) (*m/z*): [M + H]^+^ calculated for C_16_H_12_Cl_2_N_2_O, 319.0400; found, 319.0396 (Figure S10).

#### 2-Amino-5,7-dichloroquinolin-8-ol (IP-antibiotic 12)

To a solution of the amine compound 16 (80 mg, 0.25 mmol) in anhydrous DCM (3 mL), cooled to 0°C in an ice bath, boron trichloride (BCl_3_, 1 M solution in DCM, 3 equivalents) was added under argon. After 1 h of stirring in the ice bath, methanol (1 mL) was added to quench the reaction. The solvent was removed under vacuum, and the product was crystallised from the MeOH/EA/hexane to yield pure IP-antibiotic 12 (48 mg, 83%). ^1^H NMR (400 MHz, DMSO-*d*_6_): δ 6.80 (s, 2H, NH_2_), 6.94 (d, *J* = 9.1 Hz, 1H), 7.27 (s, 1H), 8.06 (d, *J* = 9.1 Hz, 1H); ^13^C NMR (101 MHz, DMSO-*d*_6_): δ 114.49, 115.51, 119.08, 119.94, 121.94, 134.04, 139.25, 146.34, 158.37; LRMS [C_9_H_6_Cl_2_N_2_O] (*m/z*): (+ve ion mode) 228.7 [M + H]^+^; HRMS (API) (*m/z*): [M + H]^+^ calculated for C_9_H_6_Cl_2_N_2_O, 228.9930; found, 228.9929 (Figure S6).

### Bacterial strains, media, and growth conditions

VRE strains ATCC 700221,^20,21^ RBWH1,^15^ GP_043 and GP_044 (kindly provided by Mark A. T. Blaskovich, The University of Queensland); *S. aureus* strains sequence type 8: MRSA USA300,^15^ VISA ATCC 700699,^22–24^ VRSA VRS1,^25,26^ and VRSA VRS4^26,27^ were cultured in brain heart infusion broth (MP Biomedicals) or cation-adjusted Mueller–Hinton broth (CAMHB) (BD Difco).

VISA 700699 was cultured in media supplemented with 4 mg/L vancomycin (Merck), while VRSA VRS1 and VRSA VRS4 were grown in media supplemented with 6 mg/L vancomycin as recommended.^28,29^ Bacterial cfu (colony forming unit) counts were performed on tryptic soy agar (BD Difco) plates. Bacteria were routinely cultured at 37°C in aerobic conditions.

### MIC assays

The MIC was defined as the minimum concentration that completely inhibited visible bacterial growth after overnight incubation at 37°C, and was determined through broth microdilution as per the CLSI guidelines.^30^ Briefly, IP-antibiotic 12 was serially diluted 2-fold across a 96-well microtitre plate in CAMHB, with the last column containing no compound as a growth control. Bacterial inoculum was prepared by direct colony suspension to approximately 2–8  ×  10^5^ cfu/mL and added to each well of the plate prior to incubation. The MIC endpoint and breakpoint of the control antibiotic nitrofurantoin are defined by CLSI.^31^ All assays were carried out in biological triplicate.

### Bacterial time-kill assays

Overnight cultures of bacteria were grown to OD_600_ = 0.5 in brain heart infusion broth at 37°C with shaking at 200 RPM before centrifugation at 3200 × *g* for 10 min at 4°C. Cells were subsequently washed with CAMHB and diluted to a starting inoculum of ∼10^5^ cfu/mL. For time-kill assays, bacteria were diluted in CAMHB, CAMHB containing IP-antibiotic 12 (1 ×  MIC), or CAMHB containing 0.8% (v/v) DMSO (Merck) only. Assays were undertaken in 96-well microtiter plates and sealed with Breathe-Easy film (ProSciTech) at 37°C for 24 h in static conditions. To quantify bacterial survival, aliquots were taken at 0, 1, 2, 4, 6, and 24 h post-treatment for enumeration via serial dilution in phosphate-buffered saline (PBS) (Gibco) before plating onto tryptic soy agar plates. Viable cfus were counted after overnight growth at 37°C. All time-kill assays were performed in biological triplicate.

### Development of resistance

Investigation of the propensity for VRE 700221, VISA 700699, and VRSA VRS1 to develop resistance against IP-antibiotic 12 in CAMHB was carried out through serial passage in sub-inhibitory concentrations of IP-antibiotic over 30 days. The antibiotic nitrofurantoin (Merck) was used for control. Initial MICs were determined via broth microdilution as per the CLSI guidelines, as previously described.^30^ The wells containing the highest concentration of IP-antibiotic or antibiotic that allowed for viable bacterial growth following overnight incubation were subsequently diluted 1:250 in CAMHB and re-inoculated into a new 96-well plate containing 2-fold dilutions of IP-antibiotic and antibiotic. This procedure was repeated over 30 days.

### Antibiotic potentiation assays

The capacity of IP-antibiotic 12 to break antibiotic resistance in VRE 700221, VISA 700699, and VRSA VRS1 was assessed using broth microdilution methodology as previously described^30^; 50 μL of standardized bacterial inoculum was added to antibiotic-embedded Sensititre^TM^ plates (ThermoFisher Scientific) in CAMHB containing sub-inhibitory IP-antibiotic 12 (0.5 ×  MIC) or CAMHB alone as per the manufacturer’s instructions. The outcome of antibiotic potentiation was assessed through the comparison of MICs after overnight static incubation at 37°C.

### Cytotoxicity

Detroit 562 cells^32^ cultured in Eagle’s minimum essential medium (American Type Culture Collection) supplemented with 10% complement inactivated fetal bovine serum (Scientifix) were washed with Hanks’ Balanced Salt Solution (Merck) before treatment with Eagle’s minimum essential medium containing IP-antibiotic 12, PBT2 (2, 4 or 8 mg/L), 0.2% (v/v) Triton-X100 (Merck) or Eagle’s minimum essential medium alone in a microtitre plate. All reagents were pre-warmed to 37°C prior to treatment. Cells were incubated in 5% CO_2_ at 37°C, with levels of cell death quantified through measurement of LDH release from supernatant at 4- and 24-h post-treatment using the CytoTox96^®^ Non-Radioactive Cytotoxicity Assay kit (Promega) as per the manufacturer’s instructions.^33^ Statistical significance was detected by an ordinary one-way ANOVA with Dunnett’s post-test (GraphPad Prism 10).

### Isothermal titration calorimetry

ITC experiments were conducted at 25°C with continuous stirring (750 rpm) using the MicroCal PEAQ-ITC instrument (Malvern Panalytical). IP-antibiotic 12 (50 µM) and zinc (500 µM) were dissolved in 100 mM 3-(*N*-morpholino)propanesulfonic acid (MOPS) buffer (pH 7.7, 3.5% DMSO, Merck). Zinc was injected into IP-antibiotic 12, and data analysis was performed with MicroCal PEAQ-ITC analysis software and fitted to a single site-binding model.

### Inductively coupled plasma mass spectrometry

Overnight cultures of bacteria were grown to OD_600_ = 0.5 in brain heart infusion broth at 37°C with shaking at 200 RPM before centrifugation at 3200*×g* for 10 min at 4°C. Cells were harvested and resuspended in CAMHB containing media only or media supplemented with sub-inhibitory (0.5 ×  MIC) IP-antibiotic 12, PBT2 (0.5 ×  MIC) or the highest DMSO concentration equivalent and incubated for 30 min at 37°C in static conditions. Following challenge, cells were centrifuged at 3200*×g* for 10 min at 4°C, harvested, and resuspended in PBS + 0.25 M ethylenediaminetetraacetic acid (Merck). The pellet was subsequently washed thrice at 3200*×g* for 20 min at 4°C, before resuspension in PBS and re-washed an additional three times at 3200*×g* for 10 min at 4°C. A 500 μL sample was subsequently aliquoted into lysing matrix B tubes (MP Biomedicals) and stored at −20°C for later determination of protein quantification using the Pierce^TM^ Bicinchoninic Protein Assay Kit (ThermoFisher Scientific).

The remaining sample was pelleted via centrifugation as per previous wash conditions, and PBS was removed before the pellet was resuspended in 70% HNO_3_ (Ajax FineChem). Samples were subsequently transferred to a 95°C waterbath and vortexed until dissolved. Following overnight incubation at 85°C, the samples were cooled to room temperature and diluted to a final concentration of 2% HNO_3_ using UltraPure distilled water (ThermoFisher Scientific) before analysis on Agilent 7900 ICP-MS (Environmental Geochemistry Laboratory, The University of Queensland). Experiments were undertaken in biological triplicates, and data analysis was performed via one-way ANOVA with Dunnett’s multiple comparisons test (GraphPad Prism 10).

### Murine wound infection model

For wound infection, the neck area of individually housed 6-week-old BALB/c mice was shaved (*n* = 10, sex-matched), and residual hair was removed using Nair (Church & Dwight) prior to the experiment. On the day of infection, mice were anesthetised via methoxyflurane inhalation, and a small superficial wound was created on the shaved skin using a sterilized metal file. The wounds were subsequently infected with 10 μL of mid-log phase bacteria in brain heart infusion medium (2.3 × 10^6^ cfu VRSA VRS1) onto the scarified skin. Following inoculum absorption, mice were treated with topical ointment cream (Pharmacy Choice Aqueous Cream) and 100 μL of oral SSV (0.9% [w/v] sodium chloride (ThermoFisher Scientific), 0.4% [v/v] Tween 80 (Merck), 0.8% [w/v] sodium carboxymethyl cellulose (Merck), 0.5% [v/v] benzyl alcohol (Merck)), topical ointment containing 6 mM (w/w) IP-antibiotic 12, 100 μL oral linezolid (Merck) at 10 mg/kg dissolved in SSV + DMSO (10% [v/v]), or a combination of topical IP-antibiotic 12 + oral linezolid. The addition of DMSO to SSV enabled the complete solubilization of the antibiotic.

Mice were treated twice daily over 4 consecutive days and euthanized 4 days post-infection via CO_2_ inhalation. The scarified wounds were subsequently excised and homogenized in lysing matrix F tubes (MP Biomedicals) containing 1 mL PBS using the FastPrep-24 5G tissue homogenizer (MP Biomedicals) and plated onto antibiotic-selective tryptic soy agar plates (6 mg/L vancomycin) to enumerate surviving bacteria. Statistical significance was determined via one-way ANOVA with Fisher’s LSD test (GraphPad Prism 10) using log_10_-transformed values.

### Murine systemic (sepsis) infection model

For systemic infection, a 100 µL dose of 1.2–1.8 × 10^8^ cfu mid-log_10_ MRSA USA300 was injected into the IP cavity of 12- to 14-week-old, sex-matched BALB/c mice after anaesthetization via methoxyflurane inhalation. Following infection, mouse cohorts (*n* = 20) were treated via oral gavage with 100 µL SSV, SSV containing IP-antibiotic 12 (40 mg/kg) suspension or minocycline (40 mg/kg) (TCI Chemicals) solution in SSV + DMSO (10% [v/v]) at 0 and 6 h post-infection. All mice were subsequently monitored for survival over the 10 following days in the absence of further treatment. Statistically significant differences in survival rates between different treatment groups were determined by the log-rank Mantel-Cox test (GraphPad Prism 10).

### Murine lung infection model

For pulmonary infection, 12-week-old, sex-matched BALB/c mice were anaesthetized via methoxyflurane inhalation and challenged intranasally with 25 μL of 5.1 × 10^7^ cfu mid-log MRSA USA300. For treatment, mouse cohorts (*n* = 10) were orally administered a 100 μL volume of SSV, IP-antibiotic 12 (40 mg/kg) suspension in SSV, or minocycline (40 mg/kg) solution in SSV + DMSO (10% [v/v]) at 0 and 6 h post-infection. At 24 h post-infection, mice were euthanised via CO_2_ inhalation, their lungs harvested and washed in PBS. Enumeration of viable bacteria was determined through tissue homogenization in lysing matrix F tubes containing 1 mL PBS via the FastPrep-24 5G tissue homogenizer and plated onto tryptic soy agar plates supplemented with 4 mg/L oxacillin (Merck). Statistical analysis of differences in bacterial burden between treatment groups was determined via one-way ANOVA with Fisher’s LSD test test using log_10_-transformed values (GraphPad Prism 10).

## Results

### IP-antibiotic 12 exhibits direct antibacterial activity against MDR *E. faecium* and *S. aureus*

**Figure 2.**
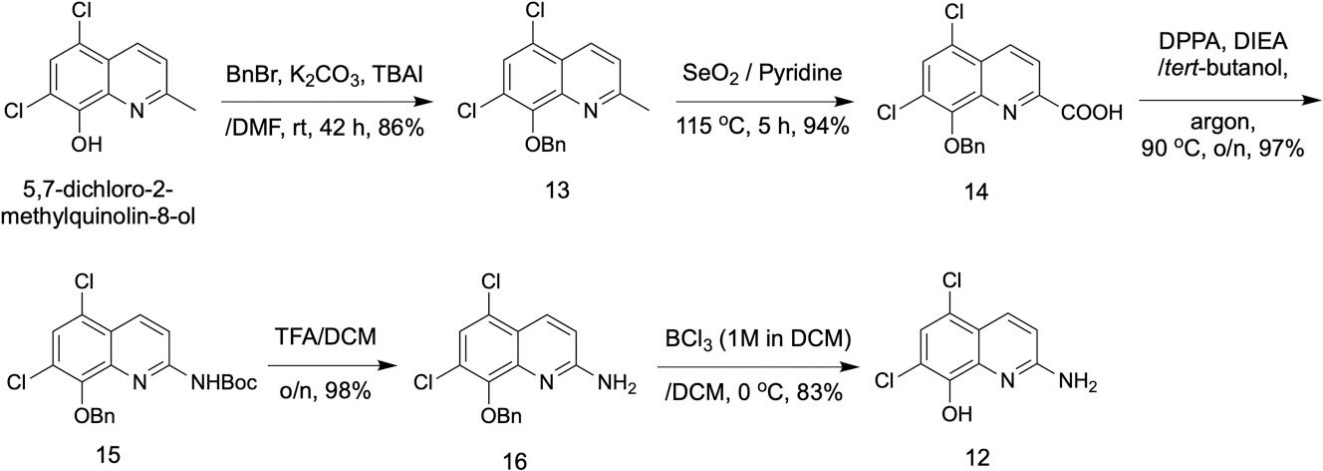
Chemical synthesis of IP-antibiotic 12. IP-antibiotic 12 was prepared following the synthetic strategy illustrated above. The synthesis started with commercially available 5,7-dichloro-2-methylquinolin-8-ol; protection of the phenolic hydroxyl group through benzyl ether formation followed by oxidation of the methyl group at position-2 affording the 2-carboxylic acid intermediate compound 14 in 94% yield. Using diphenylphosphoryl azide (DPPA); the carboxylic group of compound 14 was converted to an acyl azide which underwent a Curtius rearrangement *in situ* to form the Boc-protected amine compound 15 with 97% yield. Boc-deprotection of the amino group using trifluoroacetic acid in dichloromethane yielded the free amine compound 16 with 98% yield. Deprotection of the *O*-benzyl ether was not possible through standard catalytic reduction using palladium or other metal catalysts because of the instantaneous and strong binding of the resultant 8-HQ derivative to the metal catalyst. *O*-benzyl ether deprotection of compound 16 was achieved using boron trichloride in anhydrous dichloromethane to afford the target IP-antibiotic 12.

To assess the therapeutic antimicrobial value of a panel of IP-antibiotics (Figure 1), we first evaluated the candidates’ *in vitro* activity against *E. faecium* and *S. aureus* by minimum inhibitory concentration (MIC) testing. With the exception of candidate 11, all IP antibiotics were 8-HQ–based ionophores. In addition, our synthetic method to produce IP-antibiotic 12 was higher-yielding and utilized comparatively milder reaction conditions (Figure 2); in the original synthesis method,^19^ benzyl ether deprotection was achieved through stirring in concentrated hydrochloric acid for two nights, while in our method, deprotection was accomplished in only 1 h by stirring in borontrichloride (1 M solution in DCM) at 0°C. The overall yield of IP-antibiotic 12 starting from the carboxylic acid intermediate (compound 14, Figure 2) was 78.9% in our improved synthetic method compared to 11.4% in the original reported method.^19^

**Figure 3.**
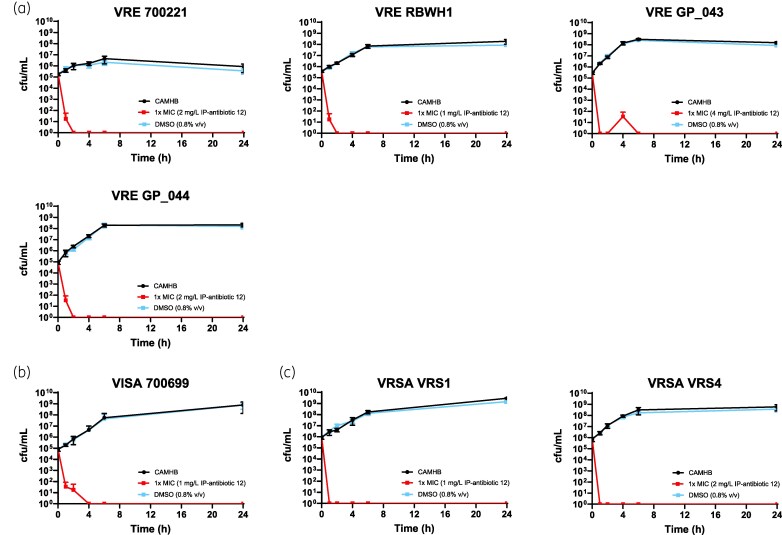
IP-antibiotic 12 is bactericidal against (a) VRE, (b) VISA and (c) VRSA. Bactericidal activity is defined as a ≥ 3-log_10_ cfu/mL reduction in inoculum size compared to the starting population.^39^ Bacteria were treated with or without IP-antibiotic 12 in CAMHB. Error bars indicate standard deviation from mean of three biological replicates.

Here, MIC analysis was performed against seven MDR strains, including vancomycin-resistant *E. faecium* (VRE), vancomycin-intermediate *S. aureus* (VISA), and VRSA. Assays were carried out in accordance with the Clinical and Laboratory Standards Institute (CLSI) guidelines.^30,31^ All employed strains (VRE strains 700221,^20,34^ RBWH1,^15^ GP_043, GP_044; VISA strain 700699;^22,35^ VRSA strains VRS1^25^ and VRS4)^26,36^ are of clinical origin. Here, compared to PBT2, the next-generation 8-HQ derivative IP-antibiotic 12 exhibited superior antimicrobial activity, with low MIC values (1–4 mg/L) observed across all tested strains (Table 1) in the absence of exogenous zinc. These values are comparable with established susceptibility breakpoints for antibiotics commonly used to treat *E. faecium* and *S. aureus* infections.^31,37^ In addition, all other IP-antibiotic candidates failed to demonstrate a comparable level of antimicrobial potency in the respective MIC screen (Table S1, available as Supplementary data at *JAC* Online). At 1 ×  MIC, IP-antibiotic 12 exhibited bactericidal activity against all strains, as confirmed by 24 h time-kill analysis (Figure 3).^38,39^

**Table 1.** IP-antibiotic 12 demonstrates potent antimicrobial activity against VRE, VISA, VRSA, and MRSA in the absence of exogenous zinc

Strain	MIC (mg/L)	
	PBT2	IP-antibiotic 12
**VRE**		
700221	>32	1–2
RBWH1	32− > 32	1
GP_043	16–32	4
GP_044	16–32	2–4
**VISA**		
700699	2	1
**VRSA**		
VRS1	16	1
VRS4	16	1–2
**MRSA**		
USA300	1–2	1

MIC assays were conducted in CAMHB, with values representing the range in MICs across three biological replicates.

### IP-antibiotic 12 exhibits a low propensity for resistance development in MDR *E. faecium* and *S. aureus in vitro*

**Figure 4.**
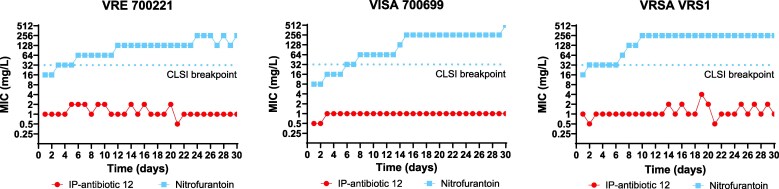
IP-antibiotic 12 demonstrates a low propensity for resistance emergence. VRE 700221, VISA 700699 and VRSA VRS1 were serially passaged with sub-inhibitory concentrations of IP-antibiotic 12 or the positive control antibiotic, nitrofuratonin for a period of 30 days. CLSI breakpoint refers to the CLSI-defined susceptibility breakpoint of nitrofurantoin against *E. faecium* and *S. aureus*.^31^.

*E. faecium* and *S. aureus* have a demonstrated ability to rapidly develop AMR through spontaneous mutation.^4^ To assess the potential for resistance development against IP-antibiotic 12, we serially passaged VRE 700221, VISA 700699, and VRSA VRS1 in sub-inhibitory concentrations of IP-antibiotic 12 over 30 days. Here, no strain tested exhibited appreciable or sustained increases in MIC over the course of the experiment. In contrast, MICs for the control antibiotic nitrofurantoin increased above CLSI-defined susceptibility breakpoints^31^ in all strains tested over the same time period. Occasional ≥ 4-fold increases in MIC were observed during IP-antibiotic 12 exposure; however, these changes were transient and unstable, suggesting that resistance-conferring mutations may impose a fitness cost and are not stably maintained within the heteroresistant population (Figure 4).

### IP-antibiotic 12 increases the susceptibility of VRE, VISA, and VRSA to select antibiotics at sub-inhibitory concentrations

PBT2 has been previously shown to break antibiotic resistance in MDR *E. faecium* and *S. aureus*.^15^ As such, the capacity of IP-antibiotic 12 to reverse antibiotic resistance in seven VRE, VISA, and VRSA strains was investigated. Here, in the presence of sub-inhibitory (0.5 ×  MIC) concentration of IP-antibiotic 12, resistance was broken in selected strains against daptomycin, gatifloxacin, clindamycin, linezolid, oxacillin, quinupristin/dalfopristin, trimethoprim/sulfamethoxazole, and tetracycline (Table 2).

**Table 2.** IP-antibiotic 12 reverses AMR to select antibiotics across VRE, VISA, and VRSA. MIC assays were performed in CAMHB in the presence and absence of IP-antibiotic 12 (0.5 ×  MIC)

Antibiotic class	Antibiotic	MIC (mg/L)															
		VRE 700221		VRE RBWH1		VRE GP_043		VRE GP_044		VISA 700699		VRSA VRS1		VRSA VRS4	MRSA USA300		
		CAMHB	+ IP-antibiotic 12	CAMHB	+ IP-antibiotic 12	CAMHB	+ IP-antibiotic 12	CAMHB	+ IP-antibiotic 12	CAMHB	+ IP-antibiotic 12	CAMHB	+ IP-antibiotic 12	CAMHB	+ IP-antibiotic 12	CAMHB	+ IP-antibiotic 12
Aminoglycoside	Gentamycin	>16	>16	>16	>16	>16	4	>16	>16	>16	>16	>16	16	>16	>16	>16	>16
	Streptomycin	>1000	>1000	>1000	>1000	>1000	≤1000	>1000	≤1000	>1000	≤1000	>1000	>1000	>1000	≤1000	>1000	>1000
Ansamycin	Rifampin	>4	>4	>4	>4	>4	4	>4	>4	>4	>4	>4	>4	>4	>4	>4	>4
Cephalosporin	Ceftriaxone	>64	>64	>64	>64	>64	>64	>64	>64	>64	>64	>64	>64	>64	>64	>64	>64
Cyclic lipopeptide	Daptomycin	>8	**2**	1	≤**0.25**	>8	≤**0.25**	>8	**2**	>8	>8	1	1	8	**0.5**	2	2
Fluoroquinolone	Ciprofloxacin	>2	>2	>2	>2	>2	1	>2	>2	>2	>2	>2	>2	>2	>2	>2	>2
	Gatifloxacin	>8	>8	>8	>8	>8	**<1**	>8	>8	>8	>8	>8	>8	>8	>8	>8	>8
	Levofloxacin	>8	>8	>8	>8	>8	4	>8	>8	>8	>8	>8	>8	>8	>8	>8	>8
Glycopeptide	Vancomycin	>128	>128	>128	>128	>128	32	>128	>128	64	4	>128	>128	>128	>128	<1	<1
Lincosamide	Clindamycin	>2	>2	>2	>2	>2	>2	>2	>2	>2	≤**0.12**	>2	>2	>2	>2	>2	>2
Macrolide	Erythromycin	>4	>4	>4	>4	>4	>4	>4	>4	>4	>4	>4	>4	>4	>4	>4	>4
Oxazolidinone	Linezolid	4	**1**	2	**1**	>8	**2**	4	**2**	2	1	4	**1**	4	**1**	4	**2**
β-Lactam	Ampicillin	>16	>16	>16	>16	0.5	0.5	1	1	16	>16	>16	>16	>16	>16	>16	>16
	Oxacillin + 2% NaCl	>8	>8	>8	>8	>8	**0.25**	>8	**0.5**	>8	8	>8	>8	>8	>8	>8	>8
	Penicillin	>8	>8	>8	>8	2	2	8	8	>8	>8	>8	>8	>8	>8	>8	>8
Streptogramin	Quinupristin/Dalfopristin	>4	>4	2	**0.5**	>4	>4	>4	>4	>4	>4	>4	>4	>4	>4	>4	>4
Sulfonamide	Trimethoprim/Sulfamethoxazole	>4/76	>4/76	>4/76	>4/76	>4/76	>4/76	>4/76	>4/76	1/19	0.5/9.5	>4/76	≤**1/19**	>4/76	>4/76	>4/76	>4/76
Tetracycline	Tetracycline	>16	≤**2**	>16	>16	>16	>16	>16	>16	>16	>16	8	**2**	>16	>16	>16	>16

Instances of potentiation to CLSI-defined clinical breakpoints^31^ have been highlighted in bold. Data represents the values of one biological replicate.

### IP-antibiotic 12 demonstrates a favourable toxicity profile *in vitro*

**Figure 5.**
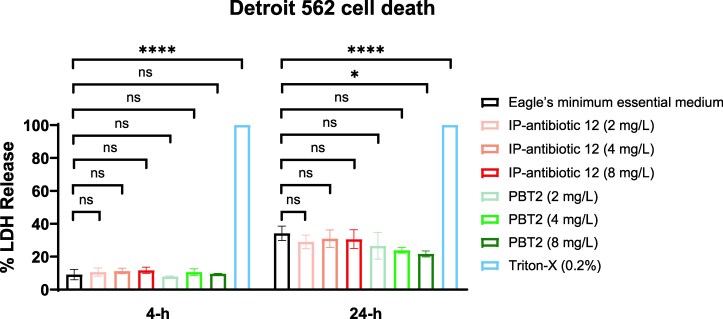
IP-antibiotic 12 demonstrates a comparable toxicity profile to its parent compound PBT2. Detroit 562 cells were co-cultured with increasing concentrations of IP-antibiotic 12, PBT2, or the positive control Trition-X in Eagle’s minimum essential medium. Cell necrosis was assessed through quantification of LDH release after 4- and 24-h incubation. Error bars represent standard deviation from mean of three biological replicates (*P < 0.05, ****P ≤ 0.0001, ordinary one-way ANOVA with Dunnett’s multiple comparisons test).

PBT2 has been shown to be safe and well-tolerated during phase I and II clinical trials.^18,40,41^ As such, we compared the cytotoxicity of IP-antibiotic 12 versus PBT2 through the measurement of lactate dehydrogenase (LDH) release in the human pharyngeal cell line Detroit 562.^42^ As a cytoplasmic enzyme, the presence of detectable LDH within culture supernatant is a biomarker for cell death.^43,44^ No significant differences in LDH release were detected between Detroit 562 cells treated with up to 8 mg/L IP-antibiotic 12 and untreated cells in Eagle’s minimum essential medium after 4 and 24 h co-culture. In addition, LDH levels of IP-antibiotic 12–treated cells were comparable to those treated with equal concentrations of the safe-for-human-use ionophore PBT2 (Figure 5).^18,40^

### IP-antibiotic 12 perturbs metal ion homeostasis in VRE, VISA, and VRSA

**Figure 6.**
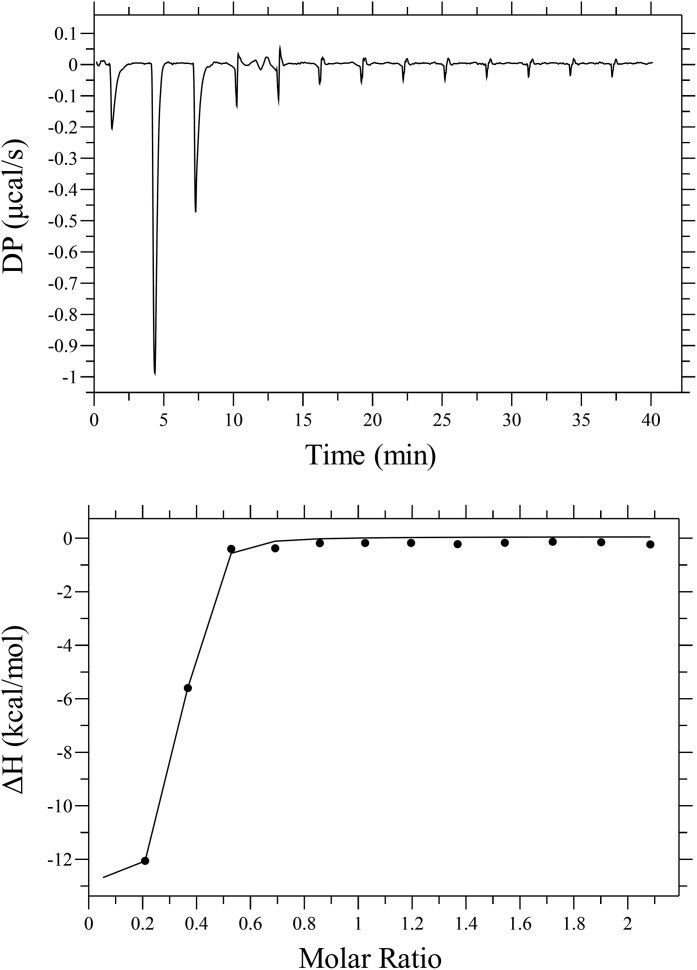
Isothermal titration calorimetry (ITC) analysis of the IP-antibiotic 12–zinc interaction. Representative titration curve and binding isotherm are representative of 3 biological replicates. In each experiment, 500 µM zinc was titrated over 12 injections into 50 µM IP-antibiotic 12 in 100 mM MOPS buffer at pH 7.7 and 25°C, with blank subtraction (Figure S1). Best-fit thermodynamic parameters were *K_D_* = 274.7 ± 92.4 nM, ΔH = -12.8 ± 0.37 kcal/mol, ΔG = −8.9 kcal/mol and −TΔS = 3.87. The fitted stoichiometry was *N* = 0.279 ± 0.0045.

**Figure 7.**
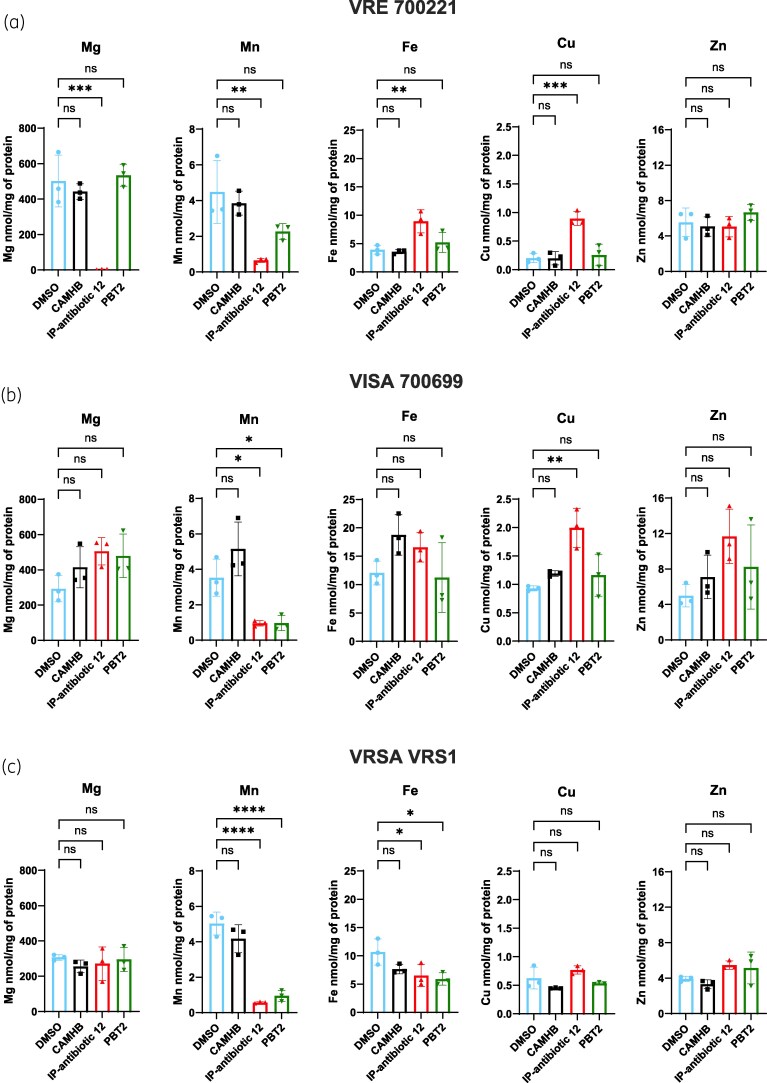
IP-antibiotic 12 dysregulates metal ion homeostasis across (a) VRE 700221, (b) VISA 700699 and (c) VRSA VRS1. Bacteria were treated in CAMHB alone or CAMHB with sub-inhibitory IP-antibiotic 12 (0.5 ×  MIC), PBT2 (0.5 ×  MIC) or DMSO (0.025% [v/v] for VRE 700221, 0.038% [v/v] for VISA 700699 and VRSA VRS1). Error bars indicate standard deviation from mean of three biological replicates (*P ≤ 0.05, **P ≤ 0.01, ***P ≤ 0.001, ****P ≤ 0.0001, one way ANOVA with Dunnett’s multiple comparisons test).

Ionophores are defined by their ability to transport metal ions across biological membranes.^14^ Here, isothermal titration calorimetry analysis demonstrated that IP-antibiotic 12 binds zinc with a binding affinity constant (*K_D_*) of 274.7 ± 92.4 nM (Figure 6, Figure S1), representing a 6-fold higher affinity than the reported zinc-binding affinity of PBT2.^45^ To investigate the impact of IP-antibiotic 12 on bacterial metal ion homeostasis, we conducted inductively coupled plasma mass spectrometry (ICP-MS) analysis following sub-inhibitory (0.5 ×  MIC) treatment. Compared to the dimethyl sulfoxide (DMSO) vehicle-carrier control, treatment with IP-antibiotic 12 led to a significant depletion in total cellular magnesium in VRE 700221, and a consistent reduction in manganese levels across all strains tested. In contrast, cellular copper levels increased in both VRE 700221 and VISA 700699. The effect on iron homeostasis appeared strain dependent, with elevated levels observed in VRE 700221 but reduced levels in VRSA VRS1. IP-antibiotic 12 did not significantly alter zinc content in either *E. faecium* or *S. aureus* strains compared to the vehicle control (Figure 7).

### IP-antibiotic 12 is efficacious in a skin disease model but ineffective in murine models of systemic and pulmonary *S. aureus* infection

**Figure 8.**
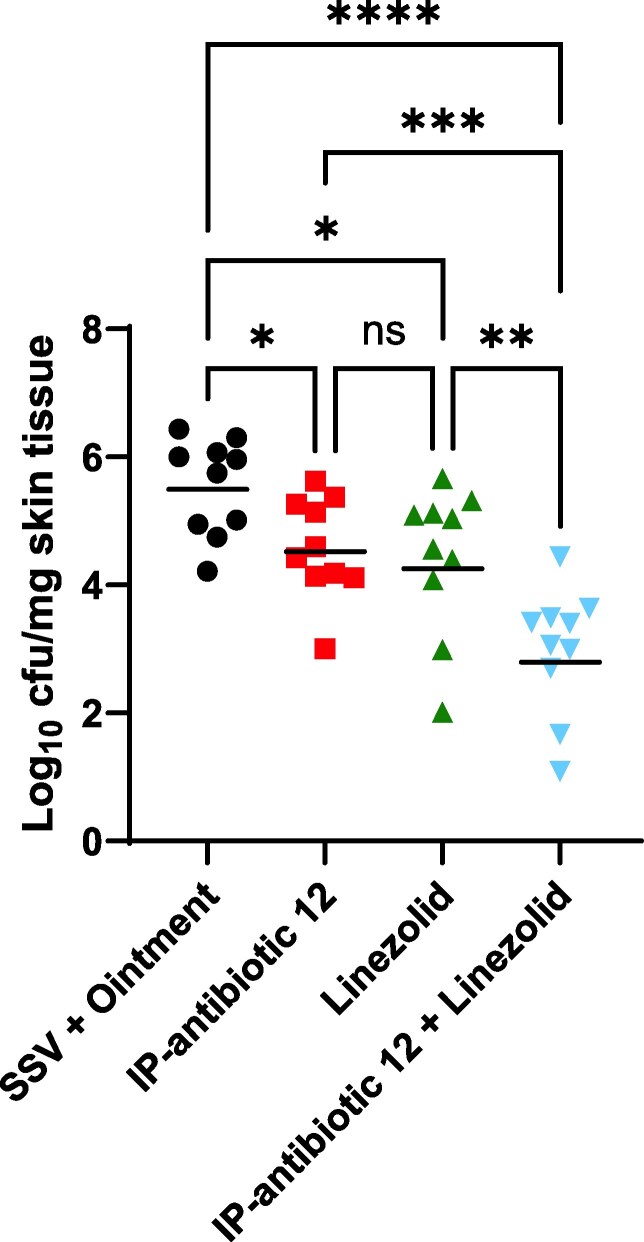
IP-antibiotic 12 acts directly and synergistically with linezolid against VRSA VRS1 in a wound infection model. Cfus were enumerated from cohorts of 6-week-old BALB/c mice (*n* = 10, sex-matched) 4 days after challenge with 2.3 × 10^6^ cfu of log-phase VRSA VRS1. Mice were housed individually and treated twice daily with oral SSV and topical ointment, topical ointment containing IP-antibiotic 12 (6 mM [w/w]), oral SSV + DMSO (10% [v/v]) containing linezolid solution (10 mg/kg) or a combination of topical IP-antibiotic 12 ointment and oral linezolid solution. The log_10_-transformed cfus are plotted for each individual mouse, and the black lines represent the geometric mean of each treatment group (*P < 0.05, **P ≤ 0.01, ****P ≤ 0.0001, ordinary one-way ANOVA with Fisher’s LSD test).

*S. aureus* is the most frequent pathogen involved in skin infections worldwide,^46^ with vancomycin-resistant strains difficult to treat due to multidrug resistance.^47^ For murine wound infection, 6-week-old BALB/c mice were scarified and infected with VRSA VRS1. Mice were subsequently treated with topical ointment cream and oral standard suspension vehicle (SSV), topical IP-antibiotic 12 (6 mM [w/v]), oral linezolid (10 mg/kg), or topical IP-antibiotic 12+ oral linezolid twice a day over 4 consecutive days before the enumeration of wounds for viable bacteria 4 days post-infection. Compared to vehicle control treated mice, topical administration of IP-antibiotic 12 resulted in a significant 1-log_10_ reduction in VRSA VRS1 bacterial burden at the site of infection (Figure 8). This reduction was statistically comparable to that achieved by oral linezolid, a standard-of-care for complicated skin infections caused by MDR *S. aureus.*^48,49^ Consistent with *in vitro* synergy data (Table 2), combination treatment with IP-antibiotic 12 and linezolid produced a significant 3-log_10_ reduction in bacterial burden compared to vehicle control (*P* ≤ 0.0001), and a 2-log_10_ reduction compared to linezolid alone (*P* ≤ 0.01). These findings suggest that IP-antibiotic 12 may be effective both as a standalone antibiotic and as an adjunct to standard therapeutic regimens for treating *S. aureus* skin and soft tissue infections. For the systemic and pulmonary infection models, the hypervirulent^50–52^ methicillin-resistant *S. aureus* (MRSA) strain USA300 was employed as VISA and VRSA strains were insufficiently virulent to establish infections in immunocompetent mice (Figures S2 and S3). However, despite low MICs (1–2 mg/L) *in vitro* (Table 1), oral administration of IP-antibiotic 12 did not provide therapeutic protection against systemic MRSA USA300 challenge (Figure S4) nor did IP-antibiotic 12 reduce bacterial lung burden compared to vehicle controls (Figure S5).

## Discussion

Metal ions such as copper, manganese, magnesium, iron, and zinc are essential for bacterial physiology, contributing to enzymatic function, DNA replication, and pathogenesis.^53–58^ To avoid metal ion toxicity or deprevation, bacterial metal ion concentrations must be tightly regulated. Disruption to this balance through either host-imposed metal sequesteration or overload is a well-established strategy of nutritional immunity.^59,60^

*E. faecium* and *S. aureus* have a highly demonstrated capacity to develop antibiotic resistance through *de novo* mutations.^4^ Thus, the inability of VRE 700221, VISA 700699, and VRSA VRS1 to develop stable, resistant mutants after 30 days of serial passage is a noteworthy finding, suggesting that IP-antibiotic 12 may have a novel mechanism of antibacterial activity and/or targets essential bacterial components that cannot be modified without significant fitness cost. Moreover, the development of resistance assay employed clinical strains with significant prior exposure to antibiotics within healthcare facilities,^61^ which can accelerate the acquisition of resistance to subsequent antibiotic treatments^62^ such as IP-antibiotic 12. To expand on this, future work may involve prolonging the development of the resistance assay and, if stable resistant mutants can be isolated, whole-genome sequencing to identify possible mutations that are associated with resistance. Subsequently, such data would help elucidate the probable bacterial targets of IP-antibiotic 12, its mechanism(s) of action, and bacterial stress response pathways.

Previous work has shown that the 8-HQ derivative PBT2 disrupts both metal ion homeostasis and oxidative stress responses in MDR *E. faecium* and *S. aureus* in the presence of zinc.^15^ We report here the first antibacterial activity study of IP-antibiotic candidate 12^19^ and its ability to perturb bacterial metal homeostasis in the absence of exogenous zinc. ICP-MS analysis of VRE 700221, VISA 700699, and VRSA VRS1 revealed strain-specific shifts in intracellular metal content. Notably, copper and iron (both capable of catalysing Fenton-like reactions) were elevated in select strains, while manganese was consistently depleted. These alterations may contribute to oxidative damage via reactive oxygen species.^63–65^ Decreased manganese may also reflect PerR-mediated activation of oxidative stress responses in *S. aureus* and enterococci.^66–71^ Broadly, disruption of metal ion homeostasis by ionophores such as PBT2 has been shown to overcome antibiotic resistance in a wide range of bacterial pathogens.^15,16,72,73^ As exemplified by VRS1, we propose that shifts in cellular metal concentrations drive both the direct antimicrobial effects of IP-antibiotic 12 and the potentiation of linezolid activity *in vivo*. The mechanism of potentiation is likely to be indirect, since linezolid acts through binding to the 30S and 50S bacterial ribosomal subunits in a process that appears independent of metal dysregulation.^74^ Nevertheless, metal homeostasis is critical for the maintenance of ribosomal structure and function.^75,76^ Thus, IP-antibiotic 12-mediated perturbation of bacterial metal homeostasis may synergize with linezolid activity through destabilization of ribosomal integrity to enhance antibiotic efficacy.

The observed intra- and inter-species variability in metal fluxes described in this study may reflect differences in bacterial transporter expression, metal binding proteins, or homeostatic systems. It is important to acknowledge that observed metal perturbations noted in this study may represent indirect bacterial responses to IP-antibiotic 12’s ionophoric activity rather than direct transport by the compound itself. Additionally, bacteria may buffer the effects through adaptive mechanisms, such as upregulating the expression of metal import/efflux systems,^77^ potentially masking significant changes in cellular content, particularly for zinc, where no measurable difference was observed.^78^ Furthermore, the use of sub-inhibitory concentrations may underestimate the full extent of IP-antibiotic 12–induced metal dysregulation. The precise molecular mechanisms by which IP-antibiotic 12 traffics metals, and the contribution of each metal to antibacterial activity, remain to be fully elucidated.

*S. aureus* is a causative agent of systemic, pulmonary, and skin infections in humans.^4^ Using immunocompetent BALB/c mice, IP-antibiotic 12 did not demonstrate antibacterial activity in *in vivo* models of systemic and lung disease but was effective as both a direct-acting and adjunct therapeutic against VRSA VRS1 wound infection. The reasons for the lack of efficacy of IP-antibiotic 12 in systemic and lung infections remain unclear but are likely related to factors such as poor aqueous solubility (∼3000 mg/L in water, > 100,000 mg/L in DMSO) and bioavailability.^14^ These challenges may be addressed in further IP-antibiotic drug discovery efforts through the development of pro-drug forms of IP-antibiotic 12 or further modifications to the 8-HQ core structure. Notably, topical administration of IP-antibiotic 12 proved efficacious in a murine model of *S. aureus* wound infection both as a direct antimicrobial and as an antibiotic potentiator. Understanding the mode of action of IP-antibiotic 12 and the mechanisms underlying bacterial resistance could further guide the design of derivatives with reduced toxicity, enhanced antimicrobial potency, and reduced likelihood of resistance development. Collectively, these findings provide a strong foundation for the development of next-generation IP-antibiotics with improved safety and efficacy *in vivo*.

## Supplementary Material

dkag186_Supplementary_Data

## Supplementary data

Figures S1 to S10 and Tables S1 and S2 are available as Supplementary data at *JAC* Online.
