# Supplementary material for: Newly identified antimicrobial activity of an 8-hydroxyquinoline-based ionophore against multidrug-resistant Enterococcus faecium and Staphylococcus aureus
Source: J Antimicrob Chemother. 2026 Jun 3;81(7):dkag186. doi: 10.1093/jac/dkag186 (PMC13232504; doi:10.1093/jac/dkag186)
Supplement: dkag186_Supplementary_Data [file dkag186_supplementary_data.docx]

Supplementary Materials

**Newly identified antimicrobial activity of an 8-hydroxyquinoline-based ionophore against multidrug-resistant *Enterococcus faecium* and *Staphylococcus aureus***

Gen LI^1,2,+,#^, Ibrahim M. EL-DEEB^3,+^, Hayden G. WHYTE^1,2^, Mark A. T. BLASKOVICH^2,4^, Mark J. WALKER^1,2,‡^, Mark VON ITZSTEIN^3,‡^, David M. P. DE OLIVEIRA^1,2,‡,*^.

^1^Australian Infectious Diseases Research Centre, Institute for Molecular Bioscience, The University of Queensland, Brisbane, QLD 4072, Australia

^2^Centre for Superbug Solutions, Institute for Molecular Bioscience, The University of Queensland, Brisbane, QLD 4072, Australia

^3^Institute for Biomedicine and Glycomics, Griffith University, Southport, QLD 4222, Australia

^4^Community for Open Antimicrobial Drug Discovery, Institute for Molecular Bioscience, The University of Queensland, QLD 4072, Australia

**^+^Contributed equally**

**‡ Equal contribution as corresponding authors**

*****Correspondence: [d.deoliveira@uq.edu.au](mailto:d.deoliveira@uq.edu.au); tel: +61 7 3365 3691 (D.M.P.D.O)

^#^Present address: A*STAR Infectious Diseases Labs (A*STAR IDL), Agency for Science, Technology and Research (A*STAR), 8A Biomedical Grove, Immunos #05-13, Singapore 138648, Singapore

**Supplementary tables and figures**

**Table S1.** MIC screen of IP-antibiotic candidates against VRE, VISA and VRSA. Data represent the range of results from 1 biological replicate.

|  | **IP-antibiotic candidate MIC (mg/L)** | | | | | | | | | | | | |
| --- | --- | --- | --- | --- | --- | --- | --- | --- | --- | --- | --- | --- | --- |
| **Strain** | **PBT2** | **1** | **2** | **3** | **4** | **5** | **6** | **7** | **8** | **9** | **10** | **11** | **12** |
|  | **VRE** | | | | | | | | | | | | |
| 700221 | > 32 | > 32 | > 32 | > 32 | > 32 | > 32 | > 32 | > 32 | > 32 | > 32 | > 32 | > 32 | 1 - 2 |
| RBWH1 | 32 - > 32 | > 32 | > 32 | > 32 | > 32 | 32 | > 32 | > 32 | > 32 | > 32 | > 32 | > 32 | 1 |
| GP_043 | 16 - > 32 | 2 | 16 - > 32 | > 32 | 32 | 16 – 32 | 4 – 8 | > 32 | > 32 | > 32 | > 32 | > 32 | 4 |
| GP_044 | 16 - > 32 | 2 | 16 - > 32 | > 32 | 16 | 32 - > 32 | 8 | 4 | > 32 | > 32 | 32 - > 32 | > 32 | 2 - 4 |
|  | **VISA** | | | | | | | | | | | | |
| 700699 | 2 | 0.5 - 1 | > 32 | 32 | > 32 | 32 | 16 | > 32 | > 32 | > 32 | 32 | 32 | 1 |
|  | **VRSA** | | | | | | | | | | | | |
| VRS1 | 16 | 1 | > 32 | > 32 | 32 | 32 | 16 - 32 | > 32 | > 32 | > 32 | 16 | > 32 | 1 |
| VRS4 | 16 | 1 - 2 | > 32 | 32 | 8 - 16 | 2 | 4 | > 32 | > 32 | > 32 | 32 | > 32 | 1 - 2 |

**Table S2.** Chemical structures of all tested IP-antibiotic candidates. All compounds were dissolved in 100% DMSO prior to MIC testing.

| **IP-antibiotic candidate** | **IUPAC name** | **Chemical structure** |
| --- | --- | --- |
| 1 | 8-Hydroxyquinoline |  |
| 2 | 2,8-Quinolinediol |  |
| 3 | 5,7-Dimethyl-8-quinolinol |  |
| 4 | 2-Methyl-8-quinolinol |  |
| 5 | 5,7-Dichloro-8-quinolinol |  |
| 6 | 5-Chloro-8-quinolinol |  |
| 7 | 2-Amino-8-quinolinol |  |
| 8 | 8-Hydroxy-2-quinolinecarboxylic acid |  |
| 9 | 8-Hydroxy-2-quinolinecarboxamide |  |
| 10 | 5-Chloro-7-(morpholin-4-ylmethyl)quinolin-8-ol |  |
| 11 | 7-Hydroxyindole |  |
| 12 | 2-Amino-5,7-dichloroquinolin-8-ol | 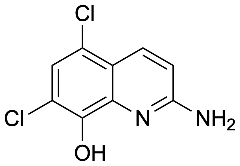 |
| PBT2 | 5,7-dichloro-2-[*N*,*N-*(dimethylamino)methyl]quinolin-8-ol | 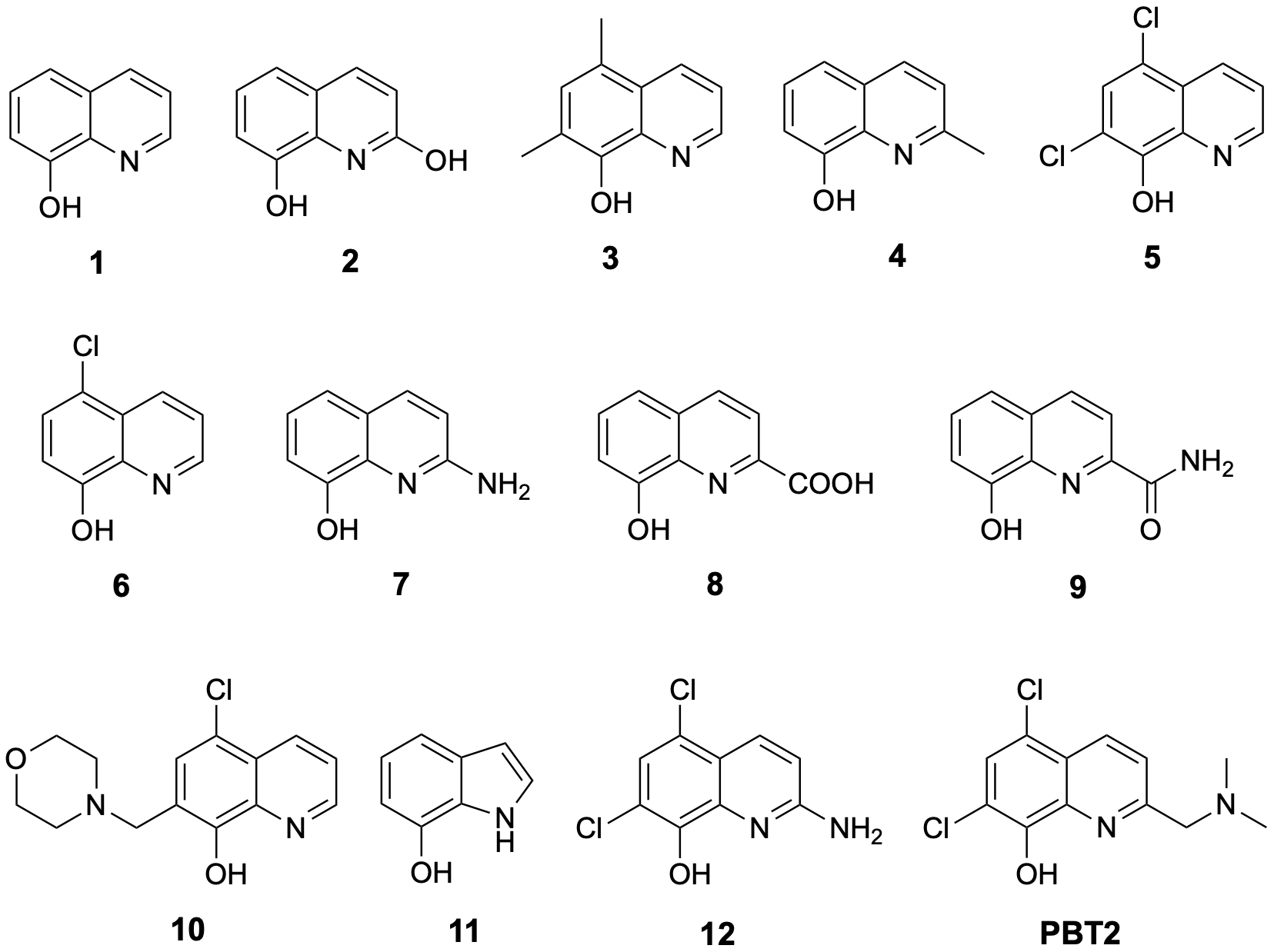 |


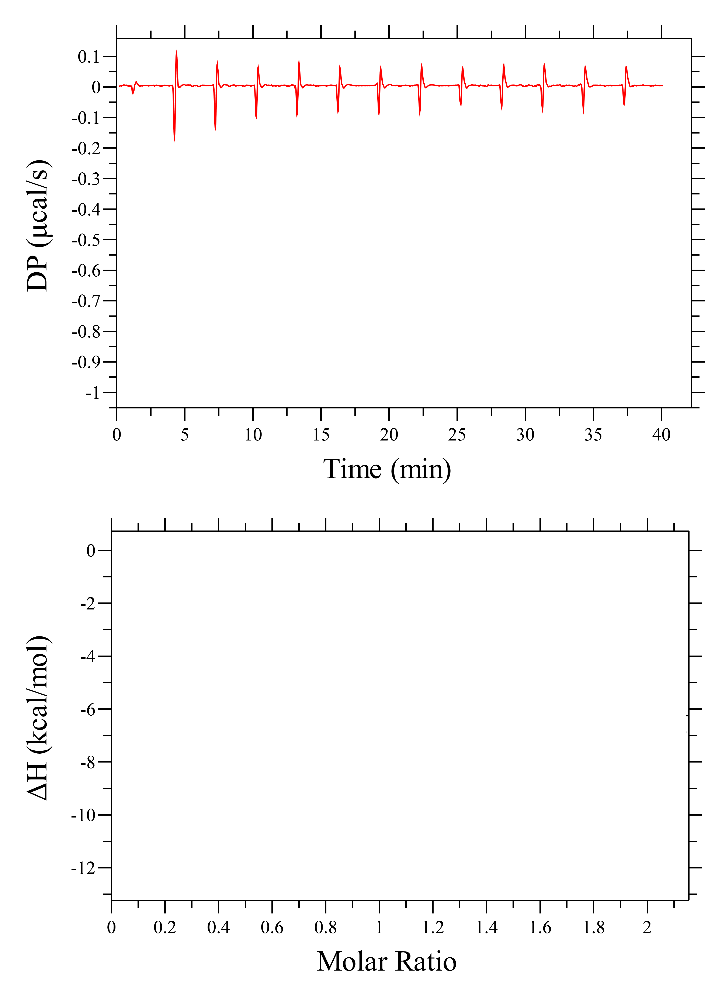


**Figure S1.** Blank isothermal titration calorimetry (ITC) experiment demonstrating negligible background signal upon injecting zinc (500 µM) into 100 mM 3-(*N*-morpholino)propanesulfonic acid buffer in the absence of IP-antibiotic 12 (pH 7.7, 25 ºC with stirring at 750 rpm). No thermodynamic parameters were obtained from the blank run, confirming that IP-antibiotic 12-zinc interactions drive the binding isotherms in Fig. 6. All ITC data in the main text are blank-subtracted.

**Figure S2.** Dose-response survival of immunocompetent BALB/c mice after intraperitoneal (I.P.) challenge with VRSA VRS1, VRSA VRS4 and VISA 700699. 12- to 14-week-old BALB/c mice (n = 10, sex-matched) were challenged with VRSA VRS1, VRSA VRS4 or VISA 700699 via I.P. injection and observed for survival for 5 days post-infection in the absence of treatment. Compared to other S. aureus strains such as MRSA USA300, the selected VRSA and VISA strains are comparatively less virulent and not lethal at inoculums of ~10^8^ cfu in this infection model (Fig. S2).

**Figure S3.** Dose-response survival of immunocompetent BALB/c mice after I.P. challenge with MRSA USA300**.** 11- to 13-week-old BALB/c mice (n = 10, sex-matched) were infected with variable concentrations of MRSA USA300 via I.P. injection and observed for survival for 5 days post-infection in the absence of treatment. In contrast to strains of VISA and VRSA (Fig. S1), MRSA USA300 was lethal at doses ~ 10^8^ cfu. The quantity of bacteria within each challenge dose only affected the percentage, but not the rate of death in infected hosts (* p ≤ 0.05, log-rank Mantel-Cox test).

**
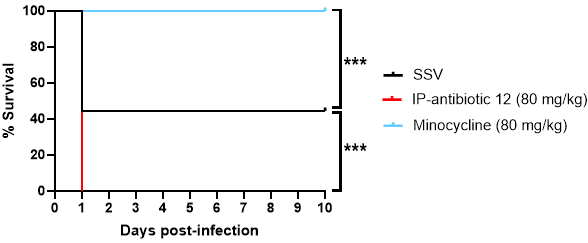
**

**Figure S4.** IP-antibiotic 12 was not protective against systemic MRSA USA300 infection***.*** 12-14-week-old immunocompetent BALB/c mice (*n* = 20, sex-matched) were challenged with 1.2-1.8 x 10^8^ cfu log-phase MRSA USA300 via IP injection followed by oral treatment with SSV only, IP-antibiotic 12 suspension in SSV (40 mg/kg) or minocycline solution (40 mg/kg) in SSV + DMSO (10% [v/v]) at 0- and 6-h post-infection. Mice were then observed for survival over the subsequent ten days in the absence of additional treatment. Mice which died on the day of infection (*n* = 2) have been excluded from results analysis. As mice in the IP-antibiotic 12 treatment group exhibited significantly more deaths than vehicle or minocycline treatment groups, under these experimental conditions, IP-antibiotic 12 is contraindicated for use against systemic MRSA USA300 infection (*** *p* ≤ 0.001, Mantel-Cox test).

**Figure S5.** Pulmonary MRSA USA300 recovery following intranasal infection and oral treatment in immunocompetent BALB/c mice*.* Administration of IP-antibiotic 12 in 12-week-old BALB/c mice (*n* = 10, sex-matched) was non-superior in reducing MRSA USA300 pulmonary burden compared to vehicle control at 24-h post-infection. Mice treated with minocycline demonstrated superior pulmonary bacterial clearance compared to IP-antibiotic 12 and vehicle. Mice were challenged intranasally with 5.1 x 10^7^ cfu log-phase MRSA USA300 and orally treated at 0- and 6-h post-infection with SSV only, IP-antibiotic 12 suspension in SSV (40 mg/kg) or minocycline solution (40 mg/kg) in SSV + DMSO (10% [v/v]). Each value represents the log_10_-transformed cfu from individual mice, and the black lines represent the geometric of each treatment group (* *p* < 0.05, ** *p* ≤ 0.01, ordinary one-way ANOVA with Fisher’s LSD test).

**Fig. S6: ^1^H and ^13^C NMR spectra of IP-antibiotic 12:**

**
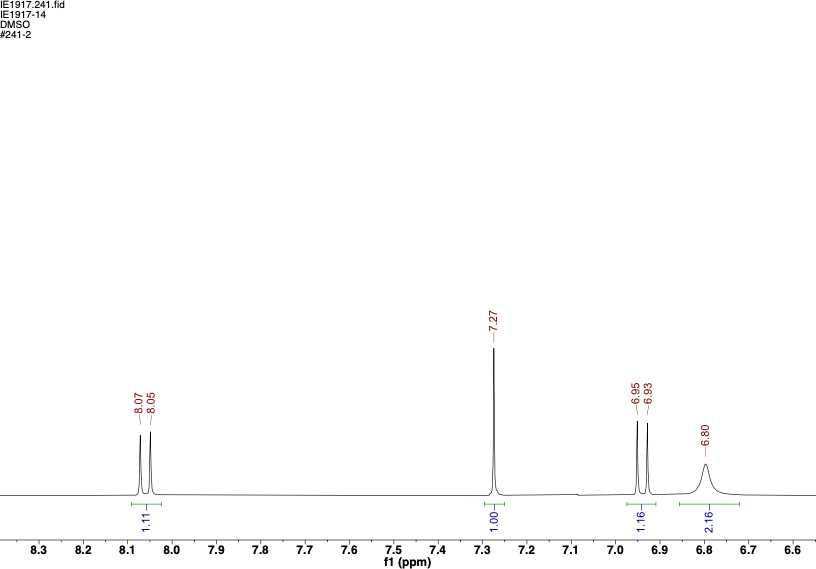

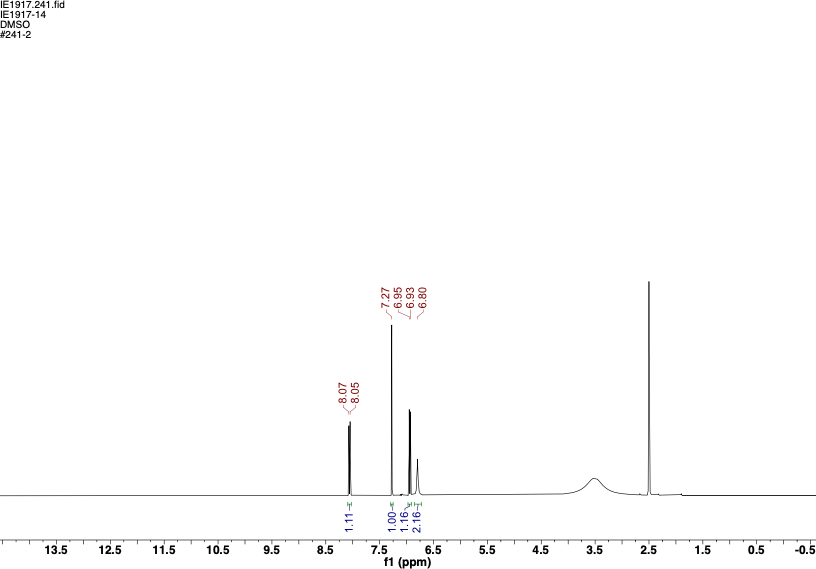
**

**^1^H NMR spectrum of IP-antibiotic 12 (400 MHz, DMSO-*d_6_*)**

**
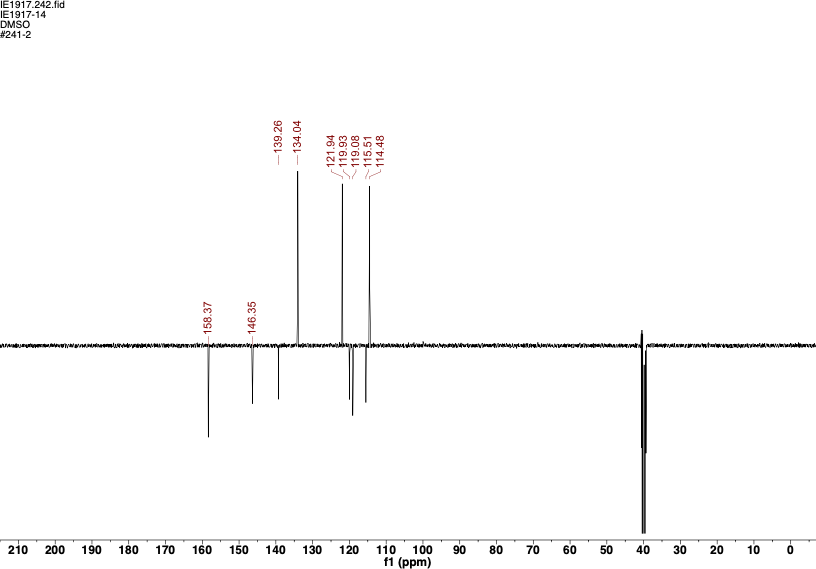
**

**^13^C NMR spectrum of IP-antibiotic 12 (101 MHz, DMSO-*d_6_*)**

Calculated Mass = 228.9930


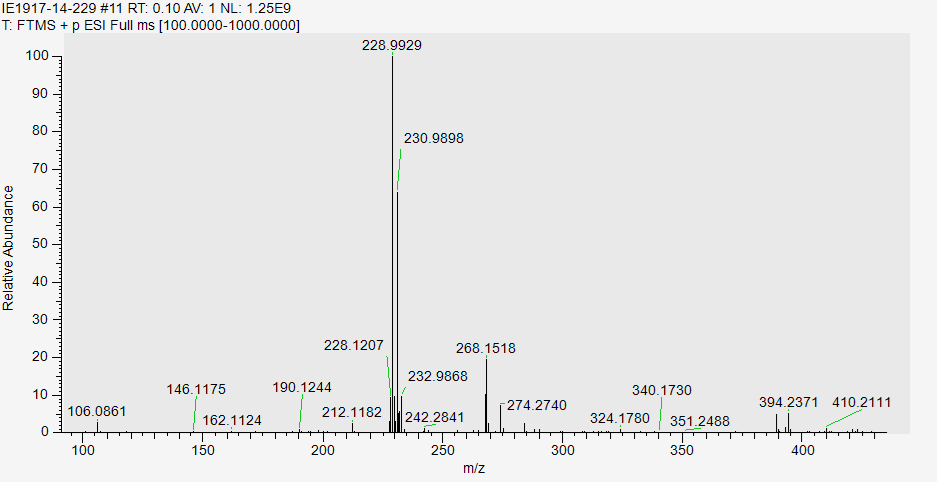


**HRMS of IP-antibiotic 12 (Positive mode)**

**Fig. S7: ^1^H and ^13^C NMR spectra of compound 13:**

**
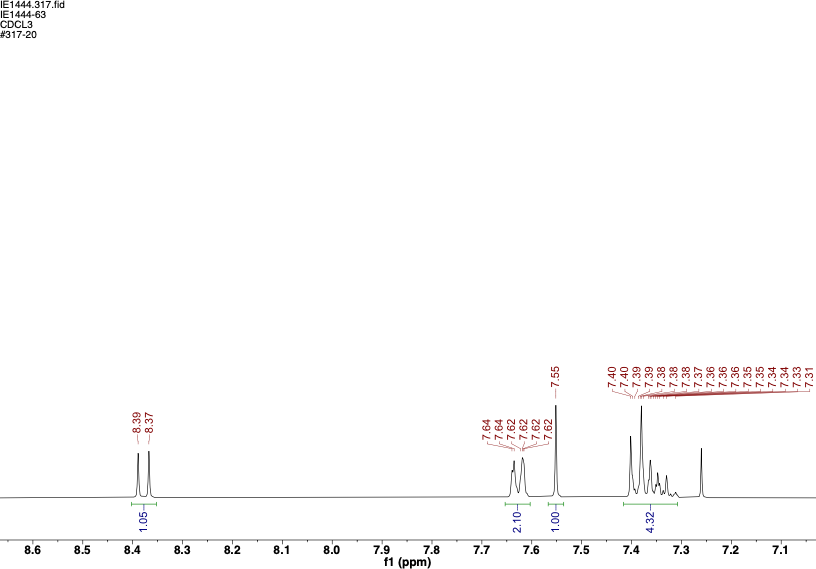

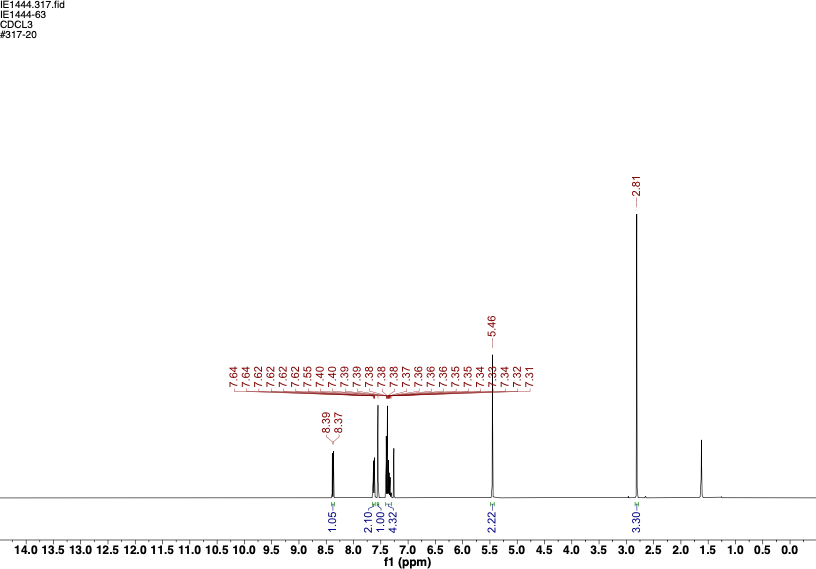
**

**^1^H NMR spectrum of compound 13 (400 MHz, CDCl_3_)**

**
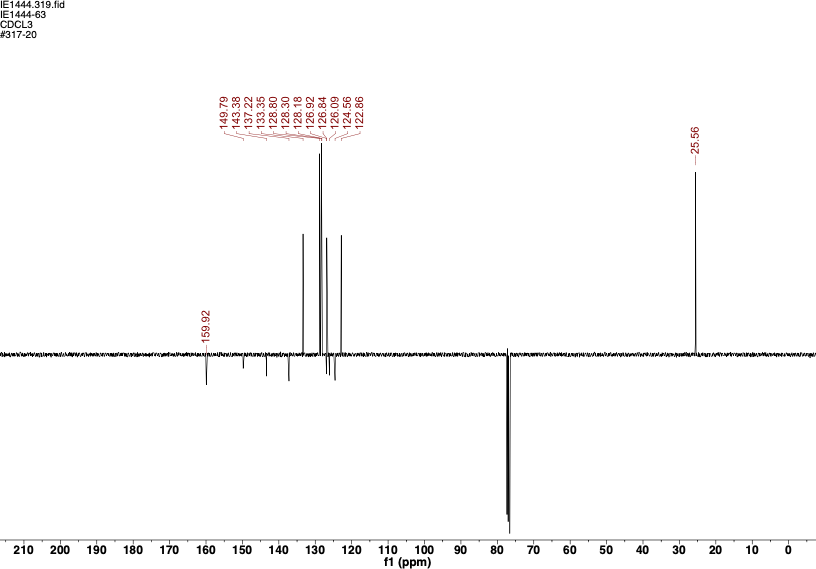
**

**^13^C NMR spectrum of compound 13 (101 MHz, CDCl_3_)**

Calculated Mass = 318.0447


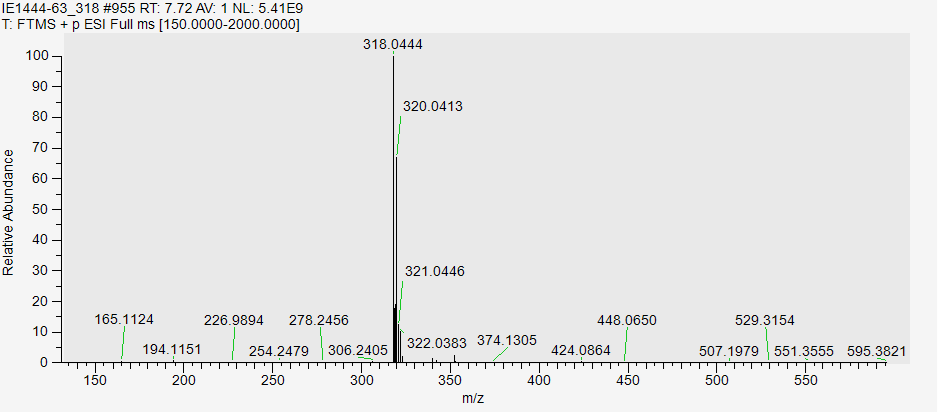


**HRMS of compound 13 (Positive mode)**

**Fig. S8: ^1^H and ^13^C NMR spectra of compound 14:**

**
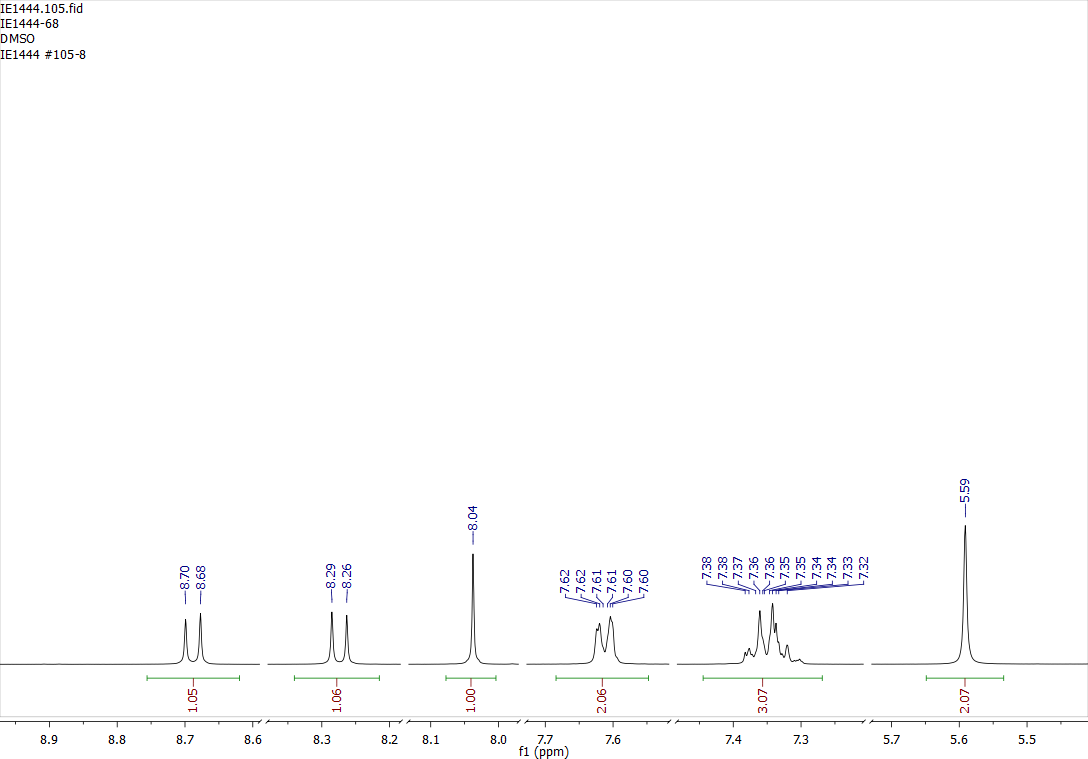

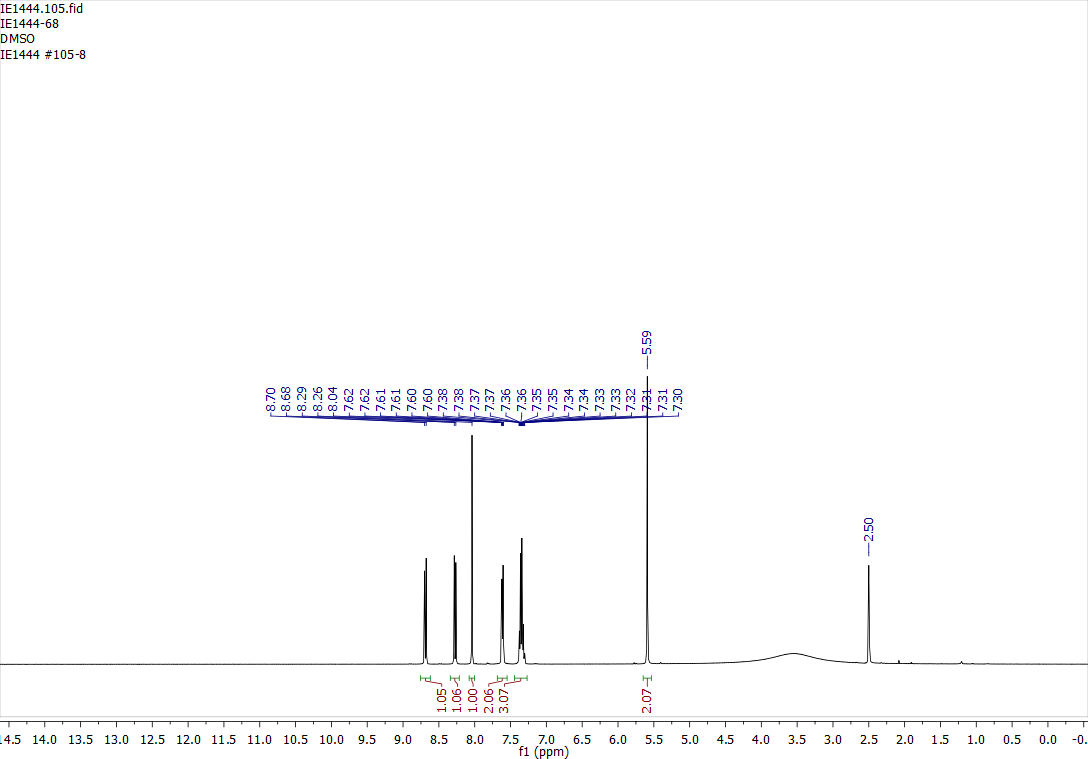
**

**^1^H NMR spectrum of compound 14 (400 MHz, DMSO)**

**
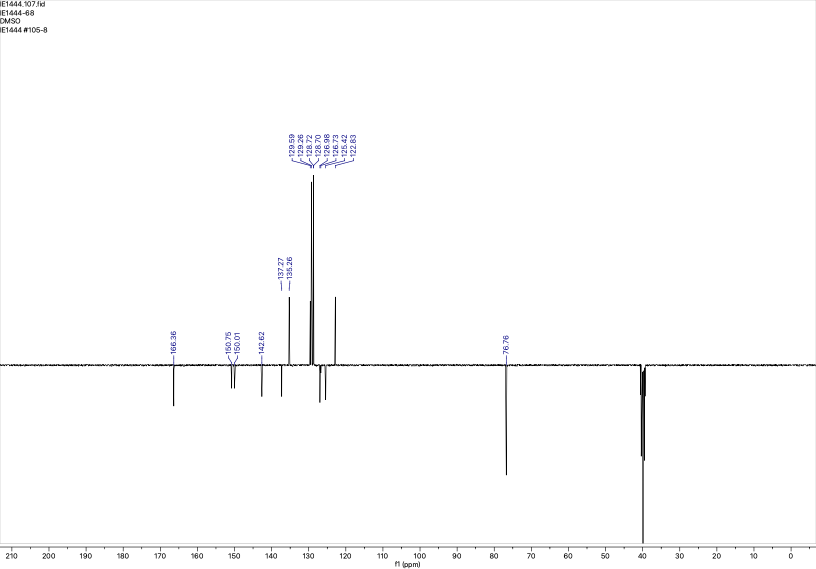
**

**^13^C NMR spectrum of compound 14 (101 MHz, DMSO)**

Calculated Mass = 348.0189


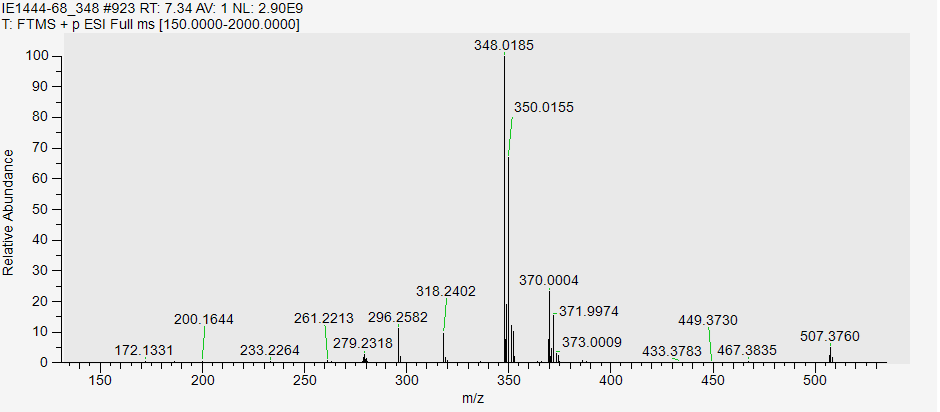


**HRMS of compound 14 (Positive mode)**

**Fig. S9: ^1^H and ^13^C NMR spectra of compound 15:**

**
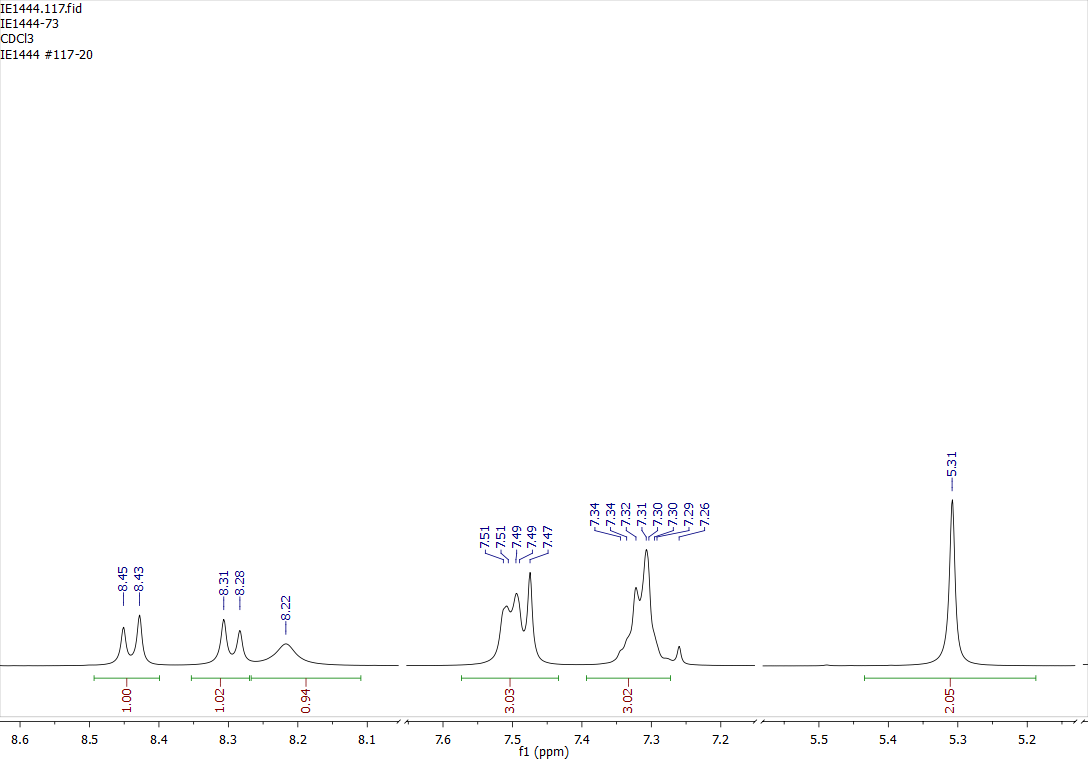

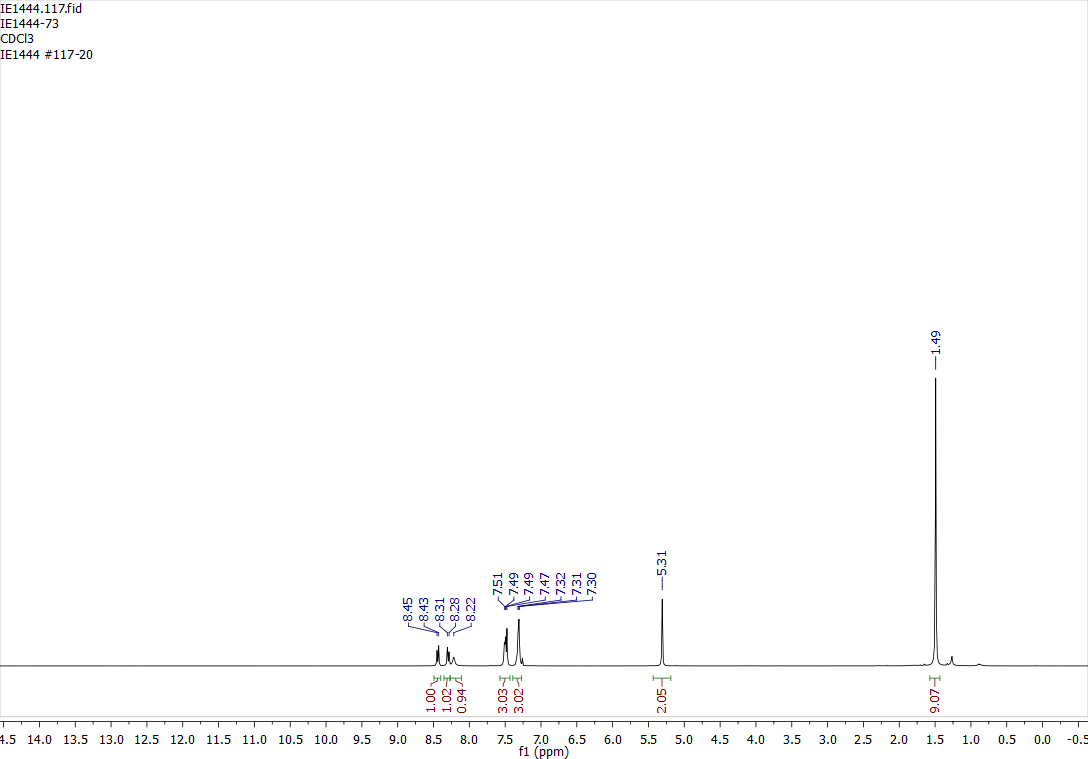
**

**^1^H NMR spectrum of compound 15 (400 MHz, CDCl_3_)**

**
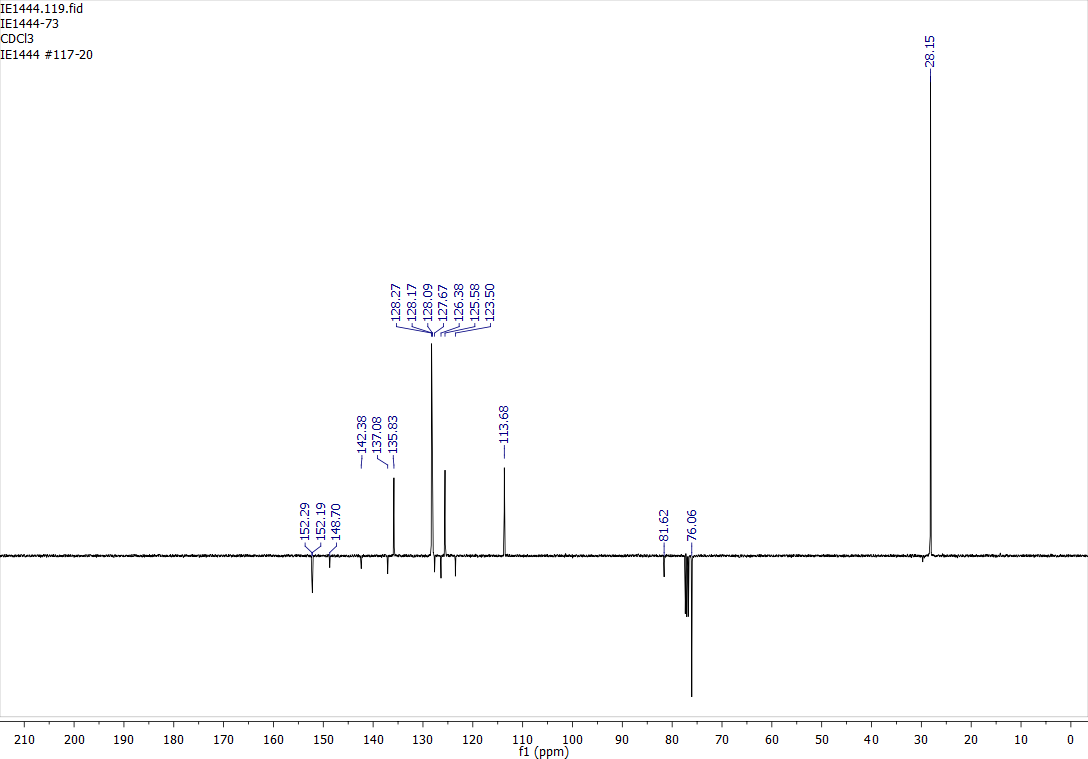
**

**^13^C NMR spectrum of compound 15 (101 MHz, CDCl_3_)**

Calculated Mass = 419.0924


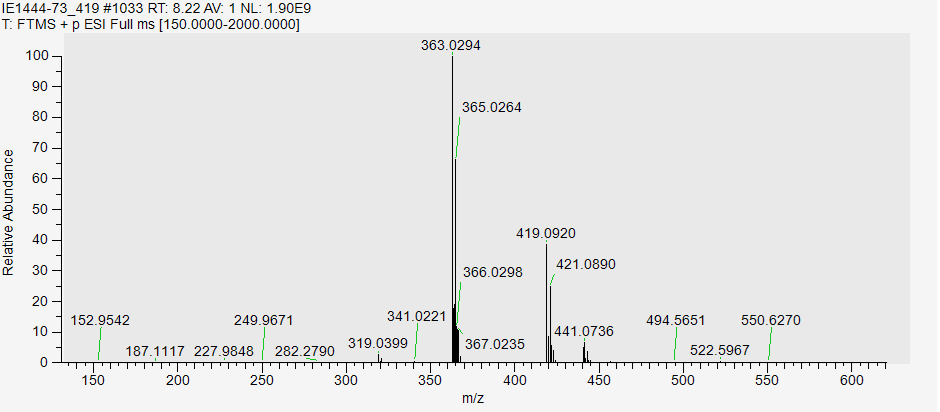


**HRMS of compound 15 (Positive mode)**

**Fig. S10: ^1^H and ^13^C NMR spectra of compound 16:**

**
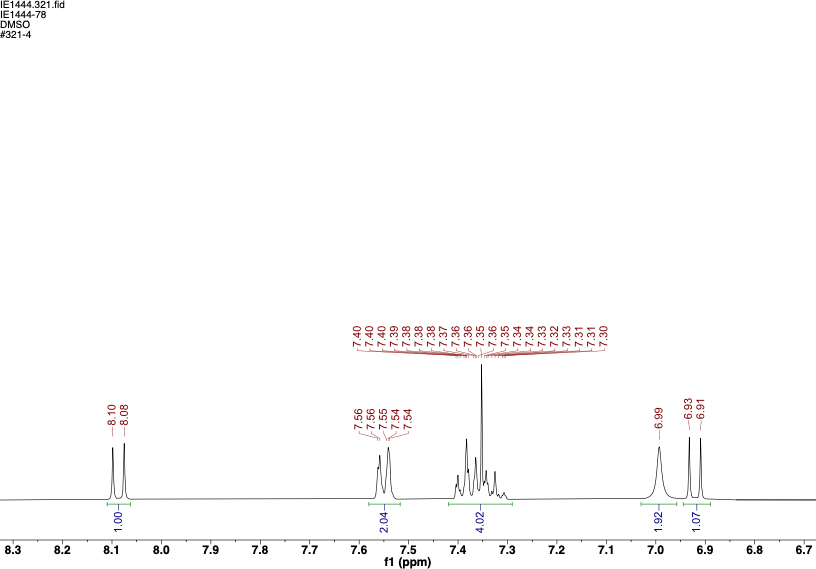

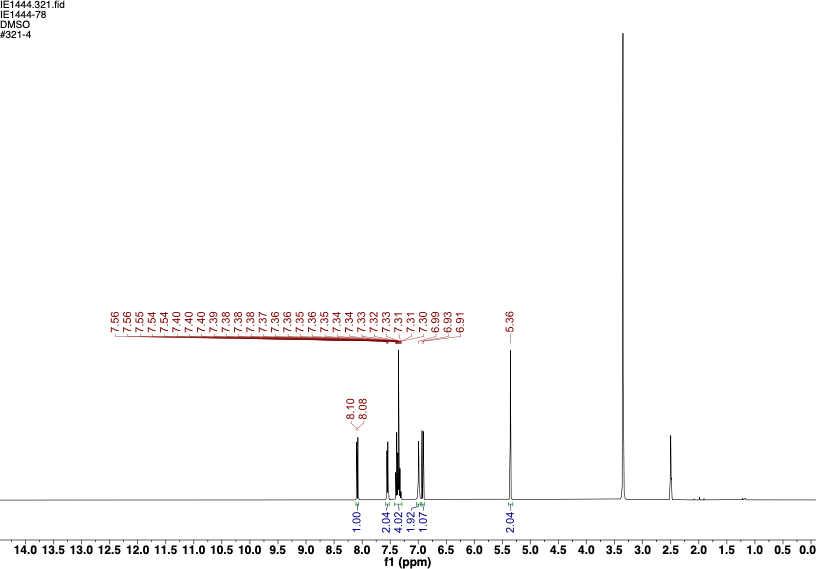
**

**^1^H NMR spectrum of compound 16 (400 MHz, DMSO)**

**
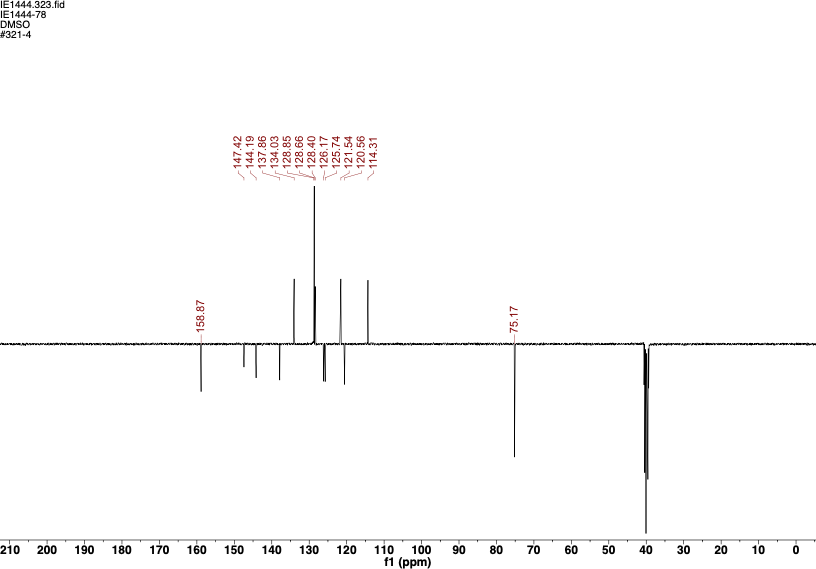
**

**^13^C NMR spectrum of compound 16 (101 MHz, DMSO)**

Calculated Mass = 319.0400


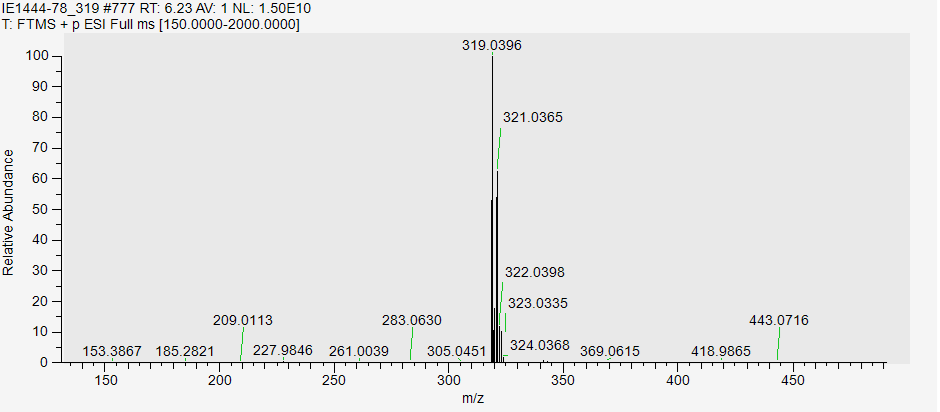


**HRMS of compound 16 (Positive mode)**
